# *IGF2BP2* maybe a novel prognostic biomarker in oral squamous cell carcinoma

**DOI:** 10.1042/BSR20212119

**Published:** 2022-02-18

**Authors:** Xiangpu Wang, Haoyue Xu, Zuo Zhou, Siyuan Guo, Renji Chen

**Affiliations:** 1 Department of Oral and Maxillofacial Plastic and Trauma Surgery, Center of Cleft Lip and Palate Treatment, Beijing Stomatological Hospital, Capital Medical University, Beijing, China; 2 Department of Oral and Maxillofacial and Head and Neck Oncology, Beijing Stomatological Hospital, Capital Medical University, Beijing, China

**Keywords:** biomarkers, IGF2BP2, immune infiltrates, OSCC, Prognosis

## Abstract

Aim: The main of the present study was to investigate the role of insulin-like growth factor 2 mRNA-binding protein 2 (*IGF2BP2*) in oral squamous cell carcinoma (OSCC) with the overarching of providing new biomarkers or potential therapeutic targets for OSCC.

Methods: We combined datasets downloaded from Gene Expression Omnibus (GEO), The Cancer Genome Atlas (TCGA), and samples collected from the clinic to evaluate the expression of *IGF2BP2* in OSCC. *IGF2BP2* survival analysis was respectively performed based on TCGA, GEO, and clinical samples. Correlations between *IGF2BP2* expression and clinicopathological parameters were then analyzed, and signaling pathways associated with *IGF2BP2* expression were identified using gene set enrichment analysis (GSEA 4.1.0). Moreover, an *IGF2BP2* co-expressed gene network was constructed, followed by gene ontology (GO) functional enrichment analysis and Kyoto Encyclopedia of Genes and Genomes (KEGG) pathway enrichment analysis on *IGF2BP2* co-expressed genes. Finally, TIMER and CIBERSORT were used to analyze the correlations among *IGF2BP2*, *IGF2BP2*-coexpressed genes, and tumor-infiltrating immune cells (TICs).

Results: *IGF2BP2* was highly expressed in OSCC and significantly correlated with overall survival of OSCC patients (*P*<0.01). High *IGF2BP2* expression correlated with poor overall survival. The GSEA results showed that cell apoptosis-, tumor-, and immune-related pathways were significantly enriched in samples with high *IGF2BP2* expression. Furthermore, GO and KEGG enrichment analyses results of *IGF2BP2* co-expressed genes indicated that these genes are mainly associated with immunity/inflammation and tumorigenesis. In addition, *IGF2BP2* and its co-expressed genes are associated with TICs (*P*<0.01).

Conclusion: *IGF2BP2* may be a potential prognostic biomarker in OSCC and correlates with immune infiltrates.

## Introduction

Oral squamous cell carcinoma (OSCC) is the eighth most common type of human cancer in the world and often has a poor prognosis. Statistics indicate that it accounts for approximately 90% of all types of oral malignancies, with over 300000 new cases and 145000 deaths every year [1]. In recent decades, the incidence and mortality of OSCC has remained at a relatively high level despite the enormous progress in diagnosis and treatments such as radiotherapy and chemotherapy. Currently, the overall 5-year survival rate of OSCC is below 60% [2]. Although the treatment methods for malignant tumors have been continuously improving from traditional surgical treatment, radiotherapy, and chemotherapy to biologically targeted therapy, the high recurrence rate and metastasis of OSCC are still not sufficiently solved and the prognosis of advanced patients is still unsatisfactory [3]. Similar to other types of tumors, the occurrence of OSCC involves a series of complex interactions between a variety of genes and proteins, which results in a multifactor interaction [4]. Therefore, this calls for elucidation of the mechanisms underlying the occurrence and development of OSCC, and the search for new specific molecular markers of OSCC with the overarching goal of facilitating development of new treatment options.

In recent years, many studies have focused on the tumor microenvironment (TME). As a complex ecosystem, TME is involved in the occurrence and development of many cancers, especially the immune components. However, studies have shown that transforming TME from tumor-friendly to tumor suppressor is a very promising new strategy for cancer treatment [5]. In addition, a previous study confirmed the correlation between immune cell infiltration and the prognosis of patients with head and neck squamous cell carcinoma (HNSC) [6]. Recently, it has been shown that immune cell dysfunction in HNSC-TME can promote immune suppression, thereby promoting the survival and progression of related tumors, and the ICI score is an effective prognostic biomarker and predictive indicator for evaluating immunotherapy response [7]. The results of Chen et al. showed that Th17 cells play a beneficial role in the prognosis of colorectal adenocarcinoma (COAD). Genes such as *KRT23, ULBP2, ASRGL1, SERPINA1*, and *SCIN* have also been identified as being related to the prognosis of Th17 cells and COAD [8]. Thus, using multilayer data analysis to identify potential immunotherapy targets and improve the therapeutic effect of OSCC has gradually become a new direction of our research.

Insulin-like growth factor 2 mRNA-binding protein 2 (*IGF2BP2*) is located in chromosome 3q27 [9]. It is a member of the *IGF2* mRNA-binding protein family. It is a post-transcriptional regulator of mRNA localization, stability and translation control. It is also a member of M6A methyltransferase-related genes [10,11]. Studies have shown that m6A modification is closely related to immune infiltration in various diseases. For example, *METTL3*-mediated m6A methylation can promote the activation of dendritic cells (DCs) on one hand [12], but on the other hand, it can destroy the balance between Treg cells and natural T cells, resulting in the destruction of their regulatory role in immune response [13]. *CDC25C, FOXM1, MCM3, MCM7* and many other key genes regulated by *IGF2BP2*-mediated RNA N6-methyladenosine are related to a variety of immune cell infiltration and tumor purity, and play an important role in the prognosis of hepatocellular carcinoma [14]. Previous studies have shown that dysregulation of *IGF2BP2* is associated with the growth, migration, adhesion, and energy metabolism of cancer cells, and it modulates the occurrence and development of many human diseases such as diabetes and malignant tumors [15,16]. *IGF2BP2* knockout mice experiments also confirmed its promotion of tumor development [17]. Studies have shown that *IGF2BP2* is associated with immune cell infiltration in esophageal cancer [18]. In addition, a recent study conducted in Taiwan reported that the genetic polymorphism of *IGF2BP2* is associated with less favorable clinical features and prognosis of patients with OSCC [19]. However, there is limited evidence regarding the association between *IGF2BP2* and OSCC, and the role of *IGF2BP2* in OSCC tumorigenesis has not yet been elucidated.

The present study analyzed the expression of *IGF2BP2* in OSCC, and the correlations between *IGF2BP2* expression and clinicopathological features, as well as prognosis using public datasets. The results were further confirmed using clinical samples and the possible molecular function of *IGF2BP2* was revealed through gene set enrichment analysis (GSEA) using data retrieved from The Cancer Genome Atlas (TCGA) database. In addition, *IGF2BP2*-related genes were screened out and used to construct gene co-expression network. Finally, the association among *IGF2BP2*, its co-expressed genes, and tumor-infiltrating immune cells (TICs) was investigated. Results obtained in the present study revealed the potential role of *IGF2BP2* in tumor immunology and its prognostic value, which will help in elucidating its possible mechanism in OSCC.

## Materials and methods

### Resources and description of public datasets

Gene Expression Omnibus (GEO) microarray series (GSE31056 [20], GSE42743 [21], and GSE51010 [22]) containing OSCC tumor and non-tumor samples were obtained from the National Center for Biotechnology Information (NCBI) (GEO, https://www.ncbi.nlm.nih.gov/geo/). All three datasets met the following inclusion criteria: (a) used human oral tissue samples; (b) had a healthy control group; and (c) contained at least 30 samples. Table 1 shows the summarized platforms and samples of GEO series.

**Table 1:** Details of GEO series included in this analysis

GEO series	Contributor(s), Year	Tumor	Non-tumor	Platform
GSE31056	Reis, 2012	22	24	GPL10526
GSE42743	Holsinger, 2012	74	29	GPL570
GSE51010	Saeed, 2013	48	8	GPL201, GPL570

All the publicly available OSCC RNA-Seq data were downloaded from TCGA’s official website (https://cancergenome.nih.gov/) using the GDC Data Transfer Tool [23]. Notably, the dataset contains survival data with clinical information and mRNA expression counts. After excluding samples with missing information, the RNA-Seq gene expression data and clinical data of 341 patients with OSCC were retained and further analyzed (Supplementary Tables S1 and S2).

### *IGF2BP2* filtering

Differentially expressed genes (DEGs) were filtered according to the log fold change (|logFC|>1) and adjusted *P* values (adj. *P*<0.001). Next, the Online Omicshare3.0 (http://www.omicshare.com/tools) was performed to discover the overlapping genes among different profiles. Finally, *IGF2BP2* was selected as the subject of the present study based on the association between the expression of overlapping genes and the prognosis of OSCC (*P*<0.01).

### Expression analysis of *IGF2BP2*

Raw CEL files of the microarray from each GEO dataset were normalized using the quantile method of Robust Multichip Analysis (RMA) from the R affy package and the normalized gene expression levels were presented as log2-transformed values by RMA [24]. *IGF2BP2* gene expression was determined by comparing tumor and non-tumor samples using the R limma package [25]. The edgeR package in R language version 3.6.3 was used to compare the mRNA expression of tumor and non-tumor samples retrieved from TCGA database [26]. Next, studies that had previously compared *IGF2BP2* expression between OSCC tumor and non-tumor samples were selected, with a threshold of *P*-value ≤ 1E-4, fold change ≥ 2, and top 10% gene rank in the Oncomine database (https://www.oncomine.org/) [27].

Furthermore, 30 pairs of OSCC tissue samples were obtained from Beijing Stomatological Hospital Affiliated to Capital Medical University from September 2019 to March 2021, followed by determination of *IGF2BP2* gene expression at the mRNA and protein level. Notably, the patients signed informed consents before the study began and the study was approved by the ethics committee of Beijing Stomatological Hospital Affiliated to Capital Medical University. Total RNA was extracted with TRIzol® reagent (Invitrogen, Carlsbad, CA, U.S.A.) and reverse transcribed to cDNA using Transcriptor First Strand cDNA Synthesis Kit (Roche, Indianapolis, IN, U.S.A.) according to the manufacturer’s instructions. Next, quantitative real-time polymerase chain reaction (qRT-PCR) was performed on the Light-Cycler96 Sequence Detection system (Roche Diagnostics, Basel, Switzerland) using SYBR® Premix ExTaq™ (Takara Bio, Inc., Otsu, Japan). Relative mRNA expression was normalized to the expression of GADPH mRNA and calculated using the the 2^−ΔΔ*C*_t_^ method. The sequences of primers used were as follows: *IGF2BP2*: forward: 5′-AGTGGAATTGCATGGGAAAATCA-3′, reverse: 5′-GTA CTC TTT GCG GTC GAG CA-3′; and *GAPDH*: forward: 5′-GGAGCGAGATCCCTCCAAAAT-3′, reverse: 5′-GGCTGTTGTCATACTTCTCATGG-3′. For Western blot analysis, protein samples were isolated using RIPA lysis buffer (Beyotime Biotechnology, Shanghai, China) containing protease inhibitor cocktail tablet (Roche Applied Science) and quantified using BCA protein assay (Beyotime Biotechnology, Shanghai, China). Next, the proteins were resolved on SDS/PAGE and transferred on to a nitrocellulose membrane. After blocking with Tris-buffered saline containing 5% skimmed milk for 1 h at room temperature, the membrane was incubated with anti-*IGF2BP2* (Abclonal Technology, Wuhan, China) and anti-GAPDH primary antibody (Abclonal Technology, Wuhan, China) overnight at 4°C. The membrane was subsequently incubated with a goat anti-mouse/rabbit secondary antibody (Boster, Wuhan, China) for 1 h at room temperature. Finally, enhanced chemiluminescence was used to visualize the protein bands in a Bio-Rad ChemiDoc XRS Imaging System (Supplementary Figures S1 and S2).

### Prognostic value of *IGF2BP2* in OSCC

Survival analysis was carried out using both survminer and survival packages in R (v.3.6.3). Eligible OSCC samples were screened in accordance with the following criteria: (i) removal of normal samples and (ii) removal of samples with incomplete clinical information. Logistic regression and the KS test were then used to analyze the correlation between the expression level of *IGF2BP2* gene and clinicopathological features of OSCC. Moreover, univariate and multivariate Cox regression analyses were performed to determine whether the prognostic significance of *IGF2BP2* was independent of the above-mentioned clinicopathological variables in OSCC. Notably, the statistical significance was tested via log-rank with the significant threshold of *P*-value set as 0.05.

### GSEA

A total of 311 OSCC tumor samples retrieved from TCGA database were divided into high and low *IGF2BP2* expression groups according to the median expression value of *IGF2BP2* [28]. GSEA 4.1.0 software was then used to determine the pathways that were enriched by the top ranked genes in the two groups: C2. CP. KEGG.v7.2 gene sets and Hallmark collections were acquired from Molecular Signatures Database (MSigDB). The number of gene set permutations for each analysis was set to 1000, and significant gene sets were determined using nominal (NOM) *P*-value <0.05 and false discovery rate (FDR) *q*-value < 0.25.

### Identification of *IGF2BP2*-related genes and construction of gene co-expression network

OSCC samples were divided into high- and low-expression groups according to the median expression value of *IGF2BP2*, and the DEGs between the two groups were analyzed using edge R package, with *P*-value <0.05 and |FC| > 1.5 cutoffs. Next, we calculated the Pearson coefficients of the DEGs and *IGF2BP2*. It is worth noting that DEGs with a *P*-value of <0.05 and a correlation coefficient of >0.3 were defined as *IGF2BP2*-related genes, and were used to construct the gene co-expression network. The top three significant genes that were positively associated with *IGF2BP2* were selected for further analysis, and their correlation with *IGF2BP2* was verified using GEIPA [29] and TIMER [30], respectively.

### Functional analyses

Gene ontology (GO) and Kyoto Encyclopedia of Genes and Genomes (KEGG) enrichment analyses were conducted using R packages clusterProfiler, enrichplot, and ggplot2 to explore the biological functions and signaling pathways of *IGF2BP2*-related genes. The significantly enriched terms were determined at *P*-value <0.05 and *q*-value <0.05.

### Correlation analysis among *IGF2BP2*, *IGF2BP2* co-expressed genes, and immune infiltrating cells

To determine the association among TICs, *IGF2BP2*, and *IGF2BP2* co-expressed genes, CIBERSORT was utilized to approximately evaluate the proportion of TICs profile in the OSCC tumor samples [31]. Furthermore, TIMER was used to verify the association among *IGF2BP2*, its co-expressed genes, and TICs. Notably, ggplot2, tidy-verse, and reshape2 packages in R version 3.6.3 software were used for analysis and plotting, and follow-up analyses were only conducted for cases with *P*-value <0.05.

### Statistical analysis

All data analyses were conducted using SPSS version 19.0 and R version 3.6.3 software. Measurement data are presented as mean ± SD. Independent-sample *t* test and paired-sample *t* test were used to analyze the differential expression levels of *IGF2BP2* mRNA between OSCC tumor tissues and non-tumor tissues retrieved from TCGA database, and samples collected at the clinic. Moreover, the association between *IGF2BP2* expression and clinicopathological characteristics was evaluated using Logistic regression and the KS test. Univariate and multivariate analyses were based on Cox proportional hazard regression models. *P*<0.05 was considered to be statistically significant.

## Results

### The analysis process of the present study

**Figure 1:**
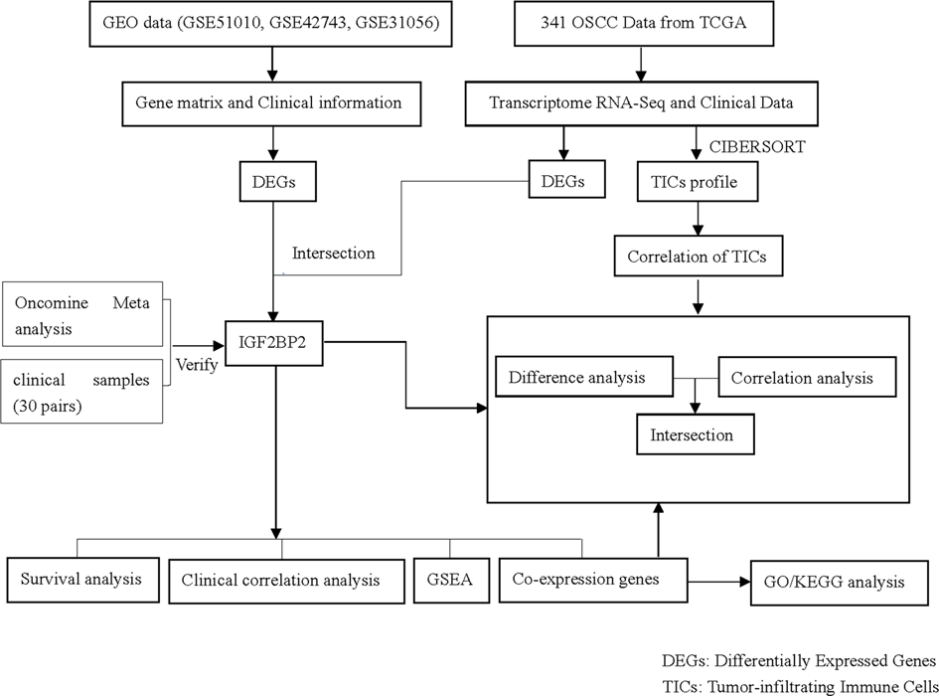
The analysis workflow of the present study

Figure 1 shows the process through which the present study was analyzed. Firstly, the OSCC gene expression datasets and corresponding clinical files were downloaded from the TCGA and GEO databases, respectively. Next, the common DEGs were screened, and *IGF2BP2* was obtained after further filtering. Furthermore, the accuracy of this result was verified through Oncomine meta-analysis and collected clinical samples. The present study focused on in-depth analysis of *IGF2BP2*, including correlation analysis of OS and clinicopathological characteristics, GSEA, co-expressed genes, and correlation with TICs. In addition, GO/KEGG enrichment analysis was performed on the co-expressed genes of *IGF2BP2*, followed by correlation analysis with TICs.

### Analysis of gene expression profiles and filtering of DEGs

**Figure 2:**
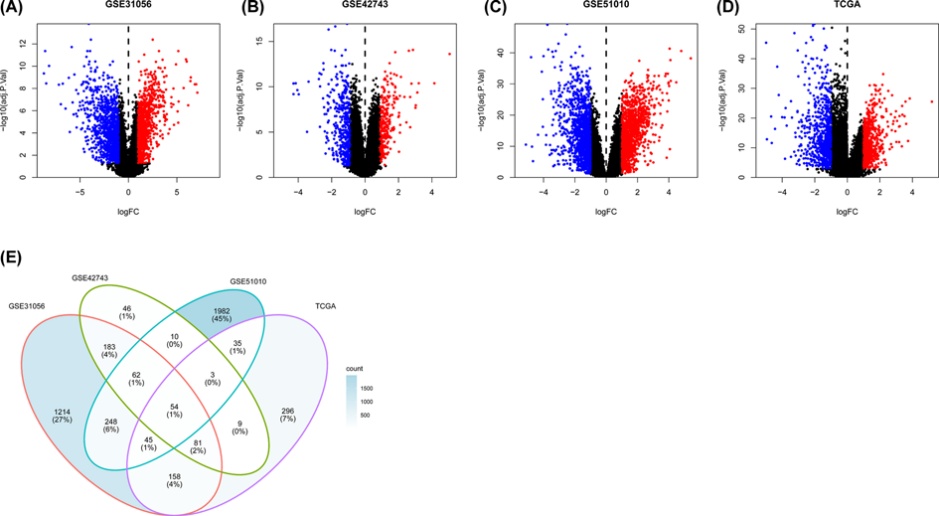
DEGs in public datasets (|logFC| > 1, *P*<0.001) (**A–C**) DEGs in GSE31056, GSE42743, GSE51010. (**D**) DEGs in TCGA. (**E**) Intersection of DEGs among GSE31056, GSE42743, GSE51010 and TCGA.

In total, 1494 DEGs, 448 DEGs, 2439 DEGs, and 1309 DEGs were screened from GSE31056, GSE42743, GSE51010, and TCGA databases, respectively (Figure 2A–D). After filtering, 54 four-crossing DEGs were identified among GSE31056, GSE42743, GSE51010, and TCGA databases (Figure 2E). Finally, *IGF2BP2* was selected for analysis in the present study after comparing the OSCC prognostic value.

### High expression of *IGF2BP2* in OSCC

**Figure 3:**
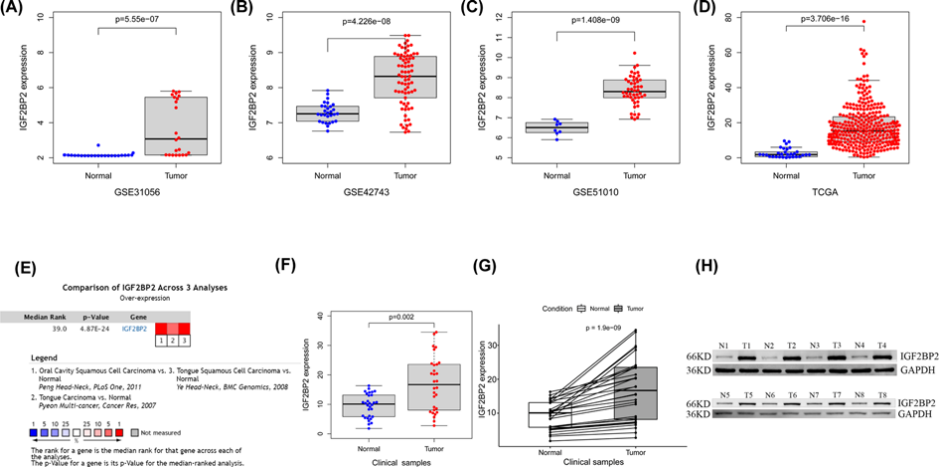
The expression level of *IGF2BP2* is up-regulated in OSCC (**A–C**) *IGF2BP2* mRNA levels in OSCC tissues and normal tissues in the GSE31056, GSE42743, and GSE51010 datasets. (**D**) *IGF2BP2* mRNA levels in OSCC tissues and normal tissues in TCGA. (**E**) Meta-analysis of *IGF2BP2* expression across three analyses in the ONCOMINE database. (**F**) mRNA expression of *IGF2BP2* based on 30 pairs of clinical samples. (**G**) *IGF2BP2* mRNA levels in OSCC tumor tissues and matched normal tissues in the 30 pairs of clinical samples. (**H**) Western blot was performed to determine the protein expression of *IGF2BP2* in OSCC tumor samples.

Results showed a significantly higher expression of *IGF2BP2* in OSCC tumor samples than in non-tumor samples in GSE31056, GSE42743, GSE51010, and TCGA datasets (Figure 3A–D, *P*<0.001). To verify the accuracy of the result, the Oncomine database was used to perform a meta-analysis of *IGF2BP2* expression in three analyses with the threshold set as *P*-value ≤1E-4, fold change ≥ 2, and top 10% gene rank. Results indicated that *IGF2BP2* was significantly up-regulated in OSCC tumor samples compared with non-tumor tissues (Figure 3E). Similar results were obtained after further verification using 30 pairs of matched clinical samples. Moreover, the results of mRNA and protein expression analysis of clinical samples showed that the expression of *IGF2BP2* was significantly higher in OSCC tumor samples than in non-tumor samples (Figure 3F–H).

### Prognostic value of *IGF2BP2* in OSCC

**Figure 4:**
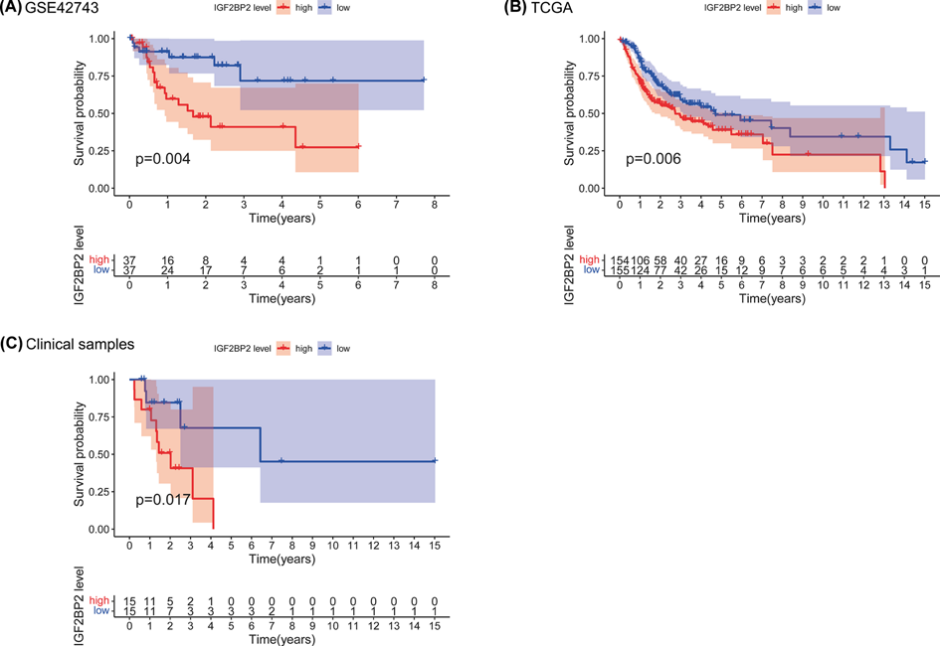
High *IGF2BP2* expression is associated with poor survival in OSCC patients (**A**) Overall survival of *IGF2BP2*^high^ and *IGF2BP2*^low^ patients analyzed with the dataset, GSE42743. (**B**) Overall survival of *IGF2BP2*^high^ and *IGF2BP2*^low^ OSCC patients analyzed with TCGA (*n*=311). (**C**) Overall survival of *IGF2BP2*^high^ and *IGF2BP2*^low^ OSCC patients analyzed with clinical samples (*n*=30).

**Figure 5:**
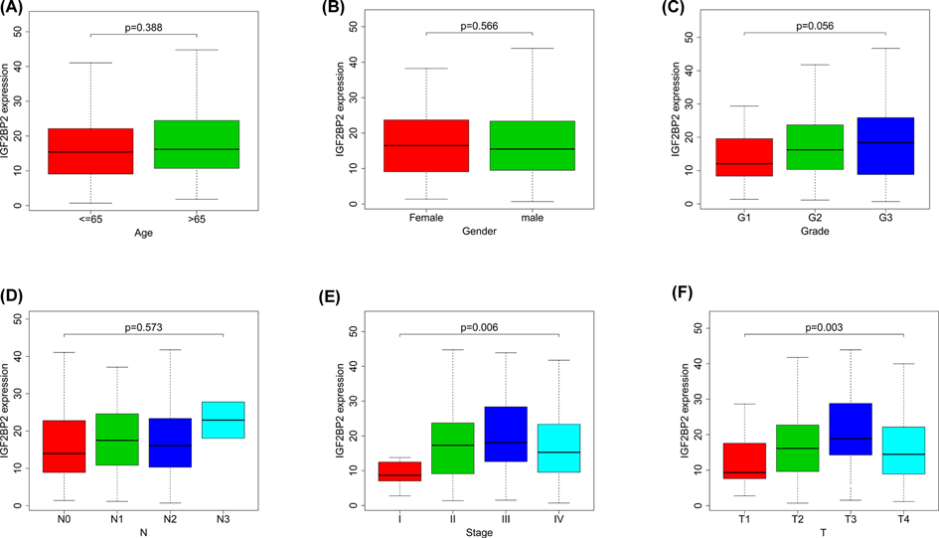
Correlation between *IGF2BP2* expression and clinicopathologic characteristics (**A**) Subgroup analysis of Age (≤65 and >65 years). (**B**) Subgroup analysis of Gender (female and male). (**C**) Subgroup analysis of Grade (G1/G2/G3). (**D**) Subgroup analysis of N stage (N0/N1/N2/N3). (**E**) Subgroup analysis of clinical stage (I/II/III/IV). (**F**) Subgroup analysis of T stage (T1/T2/T3/T4). Wilcox test in (A,B), Kruskal test in (C–F). When *P*<0.05, there was significant difference in the expression level of IGF2BP2 between subgroups with clinicopathological features.

To estimate the prognostic value of *IGF2BP2* in OSCC, Kaplan–Meier survival analysis was used to evaluate the correlation between *IGF2BP2* expression and overall survival in the TCGA and GEO datasets, and collected clinical samples, respectively. From the results, the survival of patients with high *IGF2BP2* expression was relatively poor (all *P*<0.01, Figure 4A–C). In addition, the results of the correlation analysis showed that the expression of *IGF2BP2* was significantly correlated with T stage and clinical stage, but not the N stage, grade, age, or gender (Figure 5A–F and Table 2).

**Table 2:** Correlation between the clinicopathologic characteristics and *IGF2BP2* mRNA expression (logistic regression)

Clinical characteristics	Total (*n*)	Odds ratio in *IGF2BP2* expression	*P*-value
Age (≤65 vs. >65)	310	1.027322 (0.6514473–1.620499)	0.9076
Gender (female vs. male)	311	1.113137 (0.6931055–1.790307)	0.6574
Grade (G1–2 vs. G3)	307	1.447293 (0.830286–2.547501)	0.1946
Clinical stage (I vs. II–IV)	287	2.972308 (1.102964–9.407618)	0.0418^*^
T stage (T1 vs. T2–4)	291	2.398589 (1.081688–5.721332)	0.0372^*^
N stage (N0 vs. N1–3)	266	1.276364 (0.7865679–2.076311)	0.3238

* *P*<0.05 was considered statistically significant.

### *IGF2BP2* was an independent prognostic factor in OSCC

**Figure 6:**
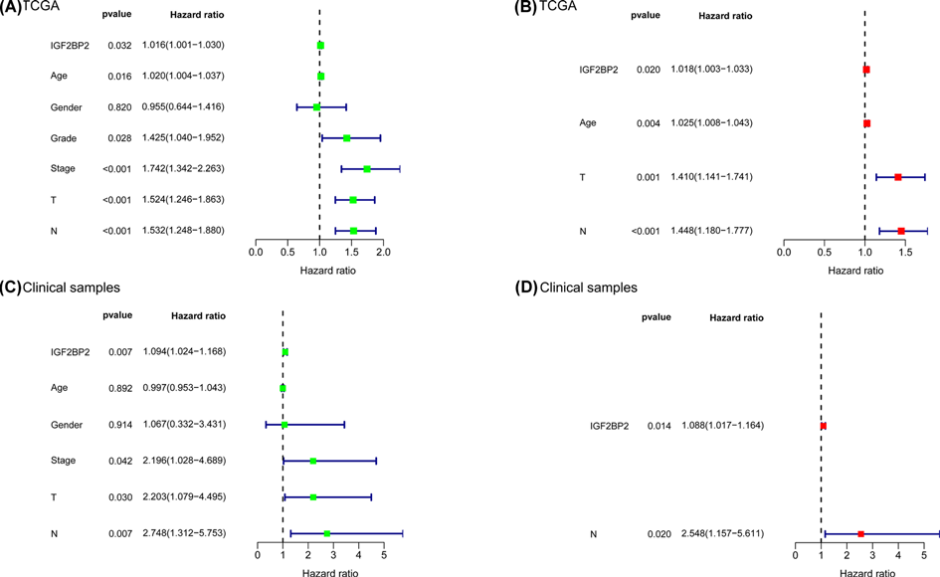
Analysis of prognostic factors for OSCC (**A**) Univariate Cox of *IGF2BP2* and six clinical phenotypes (Age, Gender, Grade, T, N, Stage) in TCGA. (**B**) Multivariate Cox of age, T, N and *IGF2BP2* in TCGA. (**C**) Univariate Cox of *IGF2BP2* and five clinical phenotypes (Age, Gender, T, N, Stage) in clinical samples. (**D**) Multivariate Cox of N and *IGF2BP2* in clinical samples.

Next, we performed univariate and multivariate cox regression analyses using the TCGA datasets and collected clinical samples, respectively. Univariate Cox regression analysis of TCGA datasets revealed that age, grade classification, clinical stage, T stage, N stage, and *IGF2BP2* were important factors for OSCC prognosis (Table 3 and Figure 6A). On the other hand, multivariate Cox regression analysis demonstrated that age, T stage, N stage, and *IGF2BP2* were independent prognostic elements for OSCC patients (Table 4 and Figure 6B). Moreover, univariate Cox regression analysis of collected clinical samples revealed that clinical stage, N stage, T stage, and *IGF2BP2* were important factors for OSCC prognosis (Supplementary Table S3 and Figure 6C), while multivariate Cox regression analysis demonstrated that N stage and *IGF2BP2* were independent prognostic elements for OSCC patients (Supplementary Table S4 and Figure 6D).

**Table 3:** Univariate Cox regression of overall survival and clinicopathologic characteristics in TCGA OSCC patients

Clinical characteristics	Hazard ratio	HR (95% CI)	*p*-value
Age (≤65 vs. >65)	1.0201	1.0036–1.0368	0.01645^*^
Gender (female vs. male)	0.9552	0.6442–1.4163	0.81960
Grade (G1/G2/G3)	1.4248	1.0400–1.9518	0.02750^*^
Clinical stage (I/II/III/IV)	1.7425	1.3420–2.2625	0.00003^*^
T stage (T1/2/3/4)	1.5236	1.2459–1.8631	0.00004^*^
N stage (N0/1/2/3)	1.5317	1.2478–1.8802	0.00004^*^
*IGF2BP2* expression (low/high)	1.0156	1.0013–1.0301	0.03151^*^

* *P*<0.05 was considered statistically significant.

**Table 4:** Multivariate analyses of overall survival and clinicopathologic characteristics in TCGA OSCC patients

Clinical characteristics	Hazard ratio	HR (95% CI)	*P*-value
Age (≤65 vs. >65)	1.0289	1.0098–1.0483	0.00284^*^
T stage (T1/2/3/4)	1.4096	1.1412–1.7419	0.00144^*^
N stage (N0/1/2/3)	1.4479	1.1798–1.7769	0.00039^*^
*IGF2BP2* expression (low/high)	1.0176	1.0028–1.0332	0.01991^*^

* *P*<0.05 was considered statistically significant.

### GSEA identified *IGF2BP2*-related signaling pathways in OSCC

**Figure 7:**
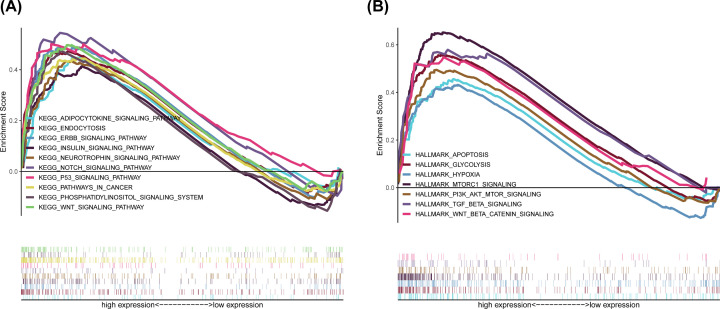
GSEA for samples with high *IGF2BP2* expression (**A**) Enriched gene sets in C2 collection, the KEGG gene sets, by samples of high *IGF2BP2* expression. Each line represents one particular gene set with unique color, and up-regulated genes are located on the left which approach the origin of the coordinates. Only gene sets both with NOM *P*<0.05 and FDR *q* < 0.25 were considered significant. Only several top gene sets are shown in the plot. (**B**) The enriched gene sets in HALLMARK collection by samples with high *IGF2BP2* expression sample.

To explore the potential molecular function of *IGF2BP2* in OSCC, GSEA was conducted between tumor samples with low and high *IGF2BP2* expression in order to predict *IGF2BP2*-related signaling pathways. Results showed that a total of 128 out of 178 signaling pathways were up-regulated, and 54 signaling pathways were significantly enriched at NOM *P*<0.05 and FDR *q*-value <0.25 (Table 5). The significantly up-regulated terms involved in tumorigenesis enriched in the high *IGF2BP2* group were ‘WNT signaling pathway’, ‘Notch signaling pathway’, ‘P53 signaling pathway’, ‘ERBB signaling pathway’, and ‘Phosphatidylinositol signaling pathway’, while the associated terms involved in immune and inflammatory responses included ‘endocytosis’, ‘insulin signaling pathway’, and ‘adipocytokine signaling pathway’ (Figure 7A). In addition, multiple immune activities and metabolic functions were respectively enriched in the *IGF2BP2* high expression group for HALLMARK gene sets (Figure 7B and Table 6). Collectively, these results suggest that *IGF2BP2* may be a promising immune-related indicator of OSCC.

**Table 5:** GSEA pathways up-regulated due to high expression of *IGF2BP2*

Gene set name	NES	NOM *P*-val	FDR *q*-val
KEGG_CELL_CYCLE	2.144	0.000	0.011
KEGG_RNA_DEGRADATION	2.101	0.000	0.011
KEGG_UBIQUITIN_MEDIATED_PROTEOLYSIS	2.007	0.000	0.011
KEGG_OOCYTE_MEIOSIS	2.070	0.002	0.006
KEGG_SPLICEOSOME	2.096	0.002	0.007
KEGG_NUCLEOTIDE_EXCISION_REPAIR	2.058	0.002	0.007
KEGG_BASAL_TRANSCRIPTION_FACTORS	2.032	0.002	0.009
KEGG_PYRIMIDINE_METABOLISM	1.952	0.004	0.020
KEGG_WNT_SIGNALING_PATHWAY	1.838	0.004	0.034
KEGG_HOMOLOGOUS_RECOMBINATION	1.931	0.004	0.022
KEGG_PROGESTERONE_MEDIATED_OOCYTE_MATURATION	1.866	0.004	0.034
KEGG_ENDOCYTOSIS	1.846	0.004	0.034
KEGG_SMALL_CELL_LUNG_CANCER	1.851	0.004	0.034
KEGG_RNA_POLYMERASE	1.831	0.006	0.034
KEGG_BASE_EXCISION_REPAIR	1.900	0.006	0.028
KEGG_PANCREATIC_CANCER	1.806	0.006	0.039
KEGG_AMINOACYL_TRNA_BIOSYNTHESIS	1.799	0.008	0.039
KEGG_INOSITOL_PHOSPHATE_METABOLISM	1.866	0.008	0.037
KEGG_N_GLYCAN_BIOSYNTHESIS	1.864	0.008	0.032
KEGG_BLADDER_CANCER	1.615	0.008	0.079
KEGG_THYROID_CANCER	1.766	0.008	0.042
KEGG_CHRONIC_MYELOID_LEUKEMIA	1.772	0.008	0.045
KEGG_PENTOSE_PHOSPHATE_PATHWAY	1.660	0.008	0.067
KEGG_PURINE_METABOLISM	1.754	0.010	0.043
KEGG_NOTCH_SIGNALING_PATHWAY	1.769	0.010	0.042
KEGG_RENAL_CELL_CARCINOMA	1.788	0.010	0.040
KEGG_PATHWAYS_IN_CANCER	1.700	0.012	0.056
KEGG_REGULATION_OF_ACTIN_CYTOSKELETON	1.718	0.014	0.051
KEGG_CYSTEINE_AND_METHIONINE_METABOLISM	1.745	0.014	0.042
KEGG_ADHERENS_JUNCTION	1.811	0.016	0.039
KEGG_DNA_REPLICATION	1.797	0.016	0.038
KEGG_MISMATCH_REPAIR	1.753	0.016	0.042
KEGG_FRUCTOSE_AND_MANNOSE_METABOLISM	1.619	0.018	0.081
KEGG_CYTOSOLIC_DNA_SENSING_PATHWAY	1.754	0.018	0.045
KEGG_ERBB_SIGNALING_PATHWAY	1.707	0.018	0.054
KEGG_P53_SIGNALING_PATHWAY	1.722	0.018	0.051
KEGG_PROTEASOME	1.771	0.019	0.043
KEGG_GLYCOSYLPHOSPHATIDYLINOSITOL_GPI_ANCHOR_BIOSYNTHESIS	1.695	0.020	0.056
KEGG_AMINO_SUGAR_AND_NUCLEOTIDE_SUGAR_METABOLISM	1.623	0.021	0.081
KEGG_PROTEIN_EXPORT	1.681	0.023	0.061
KEGG_PATHOGENIC_ESCHERICHIA_COLI_INFECTION	1.641	0.023	0.075
KEGG_GLIOMA	1.585	0.025	0.093
KEGG_PHOSPHATIDYLINOSITOL_SIGNALING_SYSTEM	1.604	0.026	0.084
KEGG_GLYOXYLATE_AND_DICARBOXYLATE_METABOLISM	1.619	0.030	0.079
KEGG_NEUROTROPHIN_SIGNALING_PATHWAY	1.572	0.031	0.099
KEGG_ADIPOCYTOKINE_SIGNALING_PATHWAY	1.534	0.032	0.108
KEGG_ONE_CARBON_POOL_BY_FOLATE	1.677	0.033	0.061
KEGG_LONG_TERM_POTENTIATION	1.496	0.042	0.126
KEGG_INSULIN_SIGNALING_PATHWAY	1.540	0.045	0.108
KEGG_VASOPRESSIN_REGULATED_WATER_REABSORPTION	1.514	0.045	0.118
KEGG_GLYCEROPHOSPHOLIPID_METABOLISM	1.469	0.046	0.139
KEGG_EPITHELIAL_CELL_SIGNALING_IN_HELICOBACTER_PYLORI_INFECTION	1.535	0.046	0.109
KEGG_ENDOMETRIAL_CANCER	1.553	0.049	0.105
KEGG_ALANINE_ASPARTATE_AND_GLUTAMATE_METABOLISM	1.542	0.049	0.110

Abbreviation: NES, normalized enrichment score. Gene sets with NES > 1, NOM *P*-value <0.05 and FDR *q*-value <0.1 are considered as significant.

**Table 6:** The enriched gene sets in HALLMARK collection due to high expression of *IGF2BP2*

Gene set name	NES	NOM *P*-val	FDR *q*-val
HALLMARK_MITOTIC_SPINDLE	2.283	0.000	0.000
HALLMARK_UNFOLDED_PROTEIN_RESPONSE	2.229	0.000	0.000
HALLMARK_MTORC1_SIGNALING	2.180	0.000	0.000
HALLMARK_MYC_TARGETS_V1	2.160	0.000	0.001
HALLMARK_GLYCOLYSIS	2.158	0.000	0.001
HALLMARK_G2M_CHECKPOINT	2.126	0.000	0.001
HALLMARK_E2F_TARGETS	2.036	0.000	0.004
HALLMARK_PROTEIN_SECRETION	1.983	0.002	0.007
HALLMARK_MYC_TARGETS_V2	1.977	0.002	0.006
HALLMARK_DNA_REPAIR	1.967	0.002	0.006
HALLMARK_PI3K_AKT_MTOR_SIGNALING	1.845	0.004	0.021
HALLMARK_UV_RESPONSE_UP	1.747	0.004	0.043
HALLMARK_APOPTOSIS	1.747	0.006	0.039
HALLMARK_TGF_BETA_SIGNALING	1.721	0.026	0.045
HALLMARK_P53_PATHWAY	1.711	0.008	0.045
HALLMARK_WNT_BETA_CATENIN_SIGNALING	1.661	0.016	0.062
HALLMARK_APICAL_JUNCTION	1.642	0.018	0.066
HALLMARK_HYPOXIA	1.624	0.031	0.070
HALLMARK_SPERMATOGENESIS	1.506	0.046	0.128
HALLMARK_HEME_METABOLISM	1.493	0.036	0.129

Abbreviation: NES, normalized enrichment score. Gene sets with NES > 1, NOM *P*-value <0.05 and FDR *q*-value <0.1 are considered as significant.

### Analysis of genes co-expressed with *IGF2BP2* in OSCC

**Figure 8:**
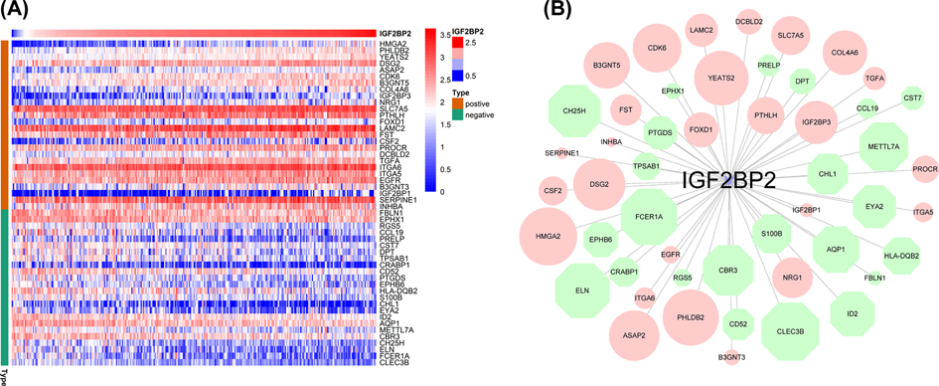
*IGF2BP2* gene co-expression network (**A**) Heatmap of *IGF2BP2* co-expression genes. The heatmap shows the top 50 genes co-expressed with IGF2BP2, including 26 positive and 24 negative genes. The row name on the right side of the heat map is gene symbol, the type on the left side of the heatmap is green, which indicates IGF2BP2 negative-related gene, and brown represents IGF2BP2 positive-related gene. (**B**) The *IGF2BP2* gene co-expression network constructed by Cytoscape version 3.8.1, red represents *IGF2BP2* positive-related genes, green represents *IGF2BP2* negative-related genes. The size of the graph drawn is proportional to the correlation of IGF2BP2.

**Figure 9:**
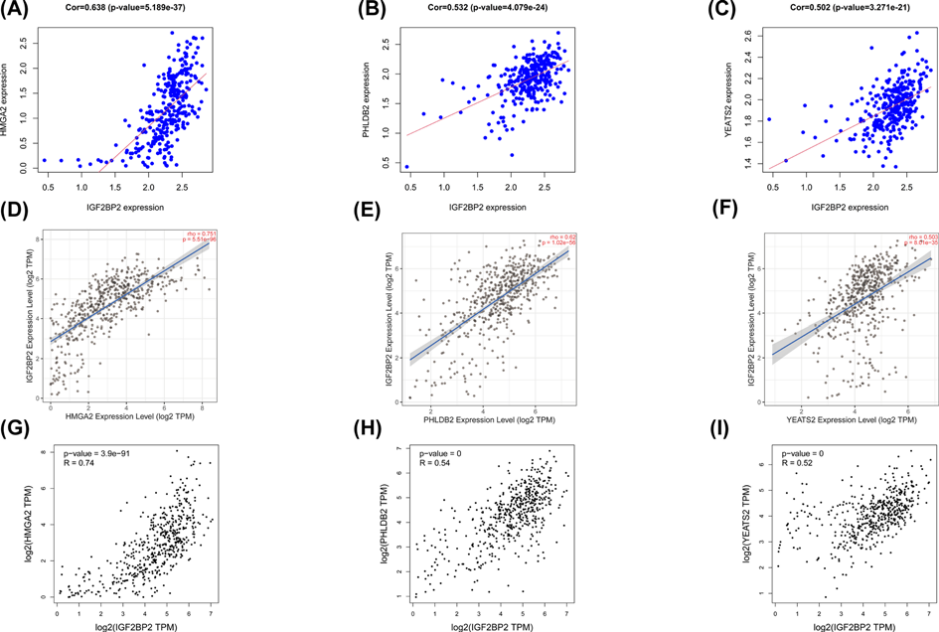
A filter of the top three significant genes that were positively associated with *IGF2BP2* (**A–C**) The genes positively associated with *IGF2BP2* in OSCC (absolute Pearson’s r ≥ 0.5) were assessed with the TCGA database. (**D–F**) *IGF2BP2* was significantly correlated with *HMGA2* (cor = 0.751, *P*=5.51e−96), *PHLDB2* (cor = 0.62, *P*=1.02e−56), *YEATS2* (cor = 0.503, *P=*8.01e−35) in OSCC (via analysis in the TIMER database). (**G–I**) *IGF2BP2* was significantly correlated with HMGA2 (cor = 0.74, *P=*3.9e−91), *PHLDB2* (cor = 0.54, *P*<0.001), *YEATS2* (cor = 0.52, *P*<0.001) in OSCC (via analysis in the GEIPA database).

**Figure 10:**
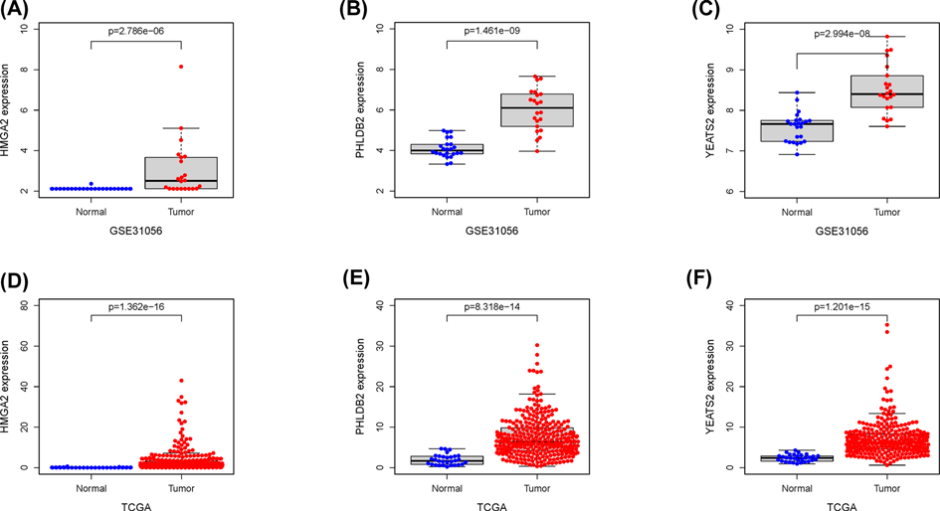
The expression of *HMGA2*, *PHLDB2* and *YEATS2* in OSCC (**A–C**) HMGA2, *PHLDB2*, and *YEATS2* mRNA levels in OSCC tissues and normal tissues in the GSE31056 dataset. (**D–F**) *HMGA2*, *PHLDB2*, and *YEATS2* mRNA levels in OSCC tissues and normal tissues in TCGA.

A total of 50 *IGF2BP2* significantly related genes were screened from the 181 DEGs of the two groups with high and low expression of *IGF2BP2*, and used to further investigate the possible effect of *IGF2BP2* in OSCC (Figure 8A). Next, the gene co-expression network was constructed using Cytoscape 3.8.1 software (Figure 8B), followed by selection of the top three significant genes that were positively correlated with *IGF2BP2* (Figure 9A–C). Furthermore, the correlation between *IGF2BP2* and these genes was verified in TIMER and GEIPA, respectively (Figure 9D–I). Results revealed that *IGF2BP2* was significantly correlated with *HMGA2* (r = 0.638, *P*=5.189e−37), *PHLDB2* (r = 0.532, *P=*4.079e−24), and *YEATS2* (r = 0.502, *P*=3.27e−21). In addition, results indicated that *IGF2BP2*-related genes were remarkably up-regulated in OSCC (Figure 10A–F). These results suggest that *IGF2BP2* and its co-expressed genes may collectively contribute to OSCC, thereby resulting in poor survival in OSCC patients.

### Functional analyses of *IGF2BP2*-related genes

**Figure 11:**
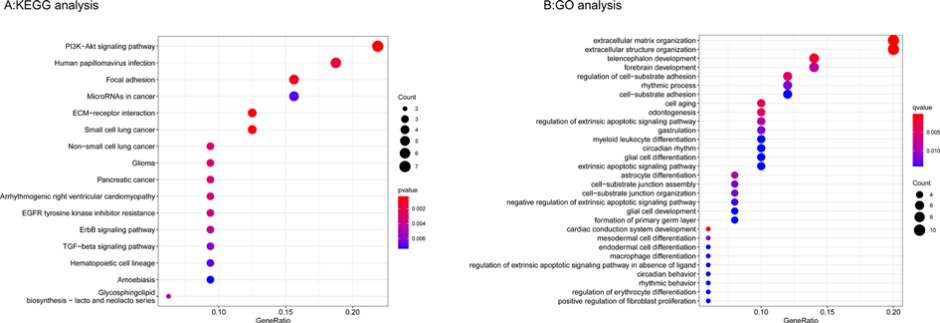
KEGG and GO biological function enrichment analyses of *IGF2BP2*-related genes (**A**) KEGG signal pathway enrichment analysis. (**B**) GO biological function enrichment analyses (when *P*-value <0.05 and *q*-value <0.05, the results were statistically significant).

KEGG pathway enrichment of *IGF2BP2*-related genes showed that ECM–receptor interaction, PI3K–Akt signaling pathway, focal adhesion, microRNAs in cancer, and Human papillomavirus infection were the most enriched pathways (Supplementary Table S5 and Figure 11A). In addition, GO analysis results proved that *IGF2BP2*-related genes were significantly enriched in regulation of the extrinsic apoptotic signaling pathway, regulation of cell–substrate adhesion, odontogenesis at BP levels; collagen containing extracellular matrix, basement membrane, and basal part of cell at CC levels; and extracellular matrix structural constituent at MF levels (Supplementary Table S6 and Figure 11B).

### *IGF2BP2* and its co-expressed genes significantly correlate with TICs in OSCC

**Figure 12:**
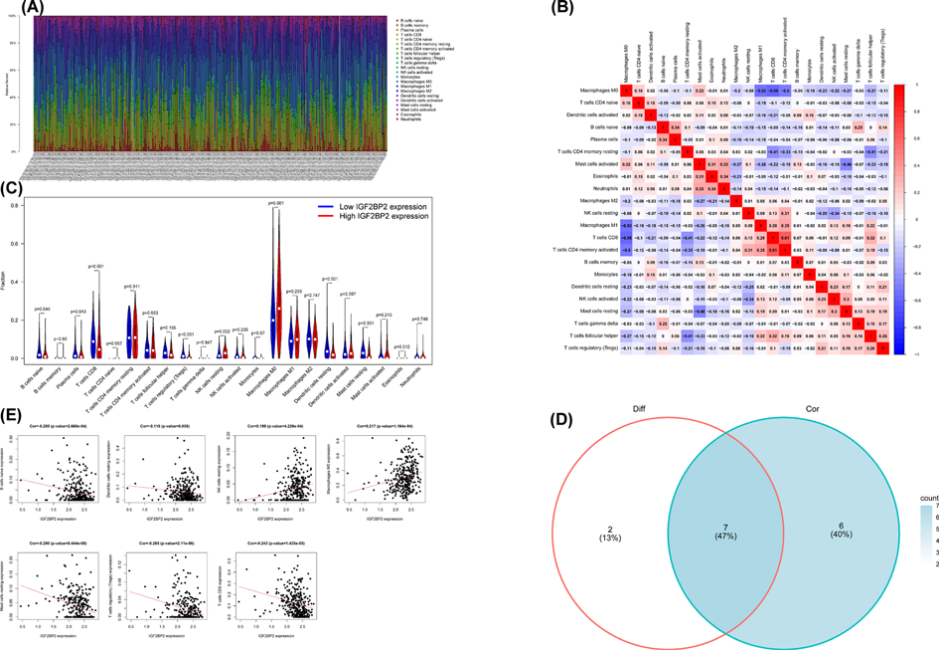
TICs profile in OSCC samples and correlation analysis, and correlation of TICs proportion with *IGF2BP2* expression (**A**) Barplot shows the proportion of 21 types of TICs in OSCC tumor samples. The row name on the right side of the figure is the name of 21 TICs, and the column name at the bottom of the figure is sample ID. (**B**) Heatmap shows the correlation between 21 kinds of TICs and numeric in each tiny box, indicating the correlation coefficient of the correlation between two cells. The shadow of each tiny color box represented a corresponding correlation value between two cells, and the Pearson’s coefficient was used for the significance test. Red represents the positive correlation between the two cells, and blue represents the negative correlation between the two cells. The darker the color, the more significant the correlation. (**C**) Violin plot showed the ratio differentiation of 21 types of immune cells between OSCC tumor samples with low or high *IGF2BP2* expression relative to the median of *IGF2BP2* expression level, and Wilcoxon rank sum was applied for the significance test. (**D**) Venn plot displayed seven kinds of TICs correlated with *IGF2BP2* expression co-determined by difference and correlation tests displayed in the violin and scatter plots, respectively. (**E**) The Scatter plot showed the correlation of seven kinds of TICs proportion with the *IGF2BP2* expression (*P*<0.05). The red line in each plot was a fitted linear model indicating the proportion tropism of the immune cell along with *IGF2BP2* expression, and the Pearson coefficient was used for the correlation test.

**Figure 13:**
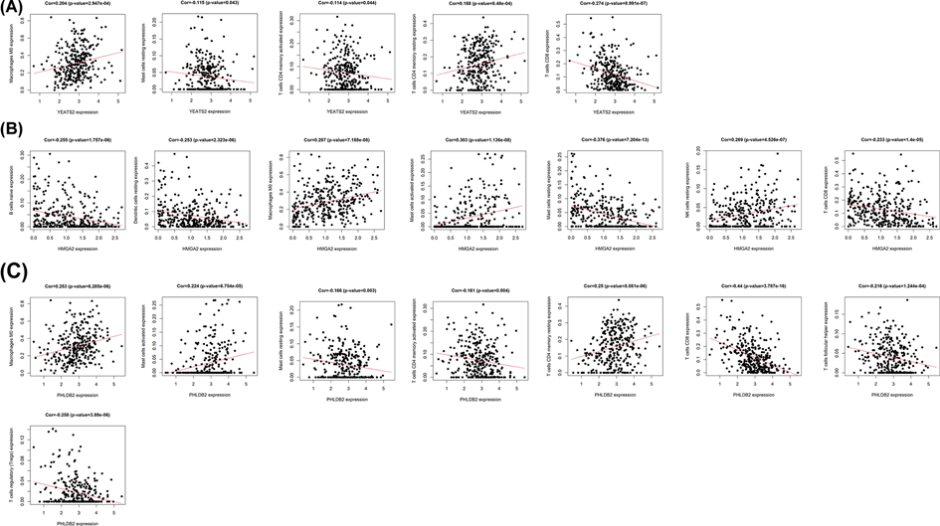
*IGF2BP2* co-expression genes were significantly correlated with the level of immunofiltration in OSCC Correlation of *YEATS2* (**A**), *HMGA2* (**B**), and *PHLDB2* (**C**) expression with TICs in OSCC.

The CIBERSORT method was applied to confirm the association between *IGF2BP2* expression and the immune component through constructing 21 types of immune cell profiles in OSCC cases and analyzing the proportion of tumor-infiltrating immune subtypes (Figure 12A–D). A total of seven kinds of TICs were found to have an association with *IGF2BP2* expression (*P*<0.001, Figure 12E). The results revealed that two TICs had a positive relationship with *IGF2BP2* expression, including resting NK cells and macrophages M0, while five kinds of TICs had a negative correlation with *IGF2BP2* expression, including naïve B cells, resting DCs, resting mast cells, CD8^+^ T cells, and regulatory T cells. Furthermore, we determined whether the *IGF2BP2* co-expressed genes (*HMGA2, PHLDB2*, and *YEATS2*) had an association with TICs (Figure 13). The above results suggest that *IGF2BP2* and its co-expressed genes may be involved in the immune response in the TME by affecting immune cells.

## Discussion

Cancer has become one of the most important cause of death among middle-aged and elderly people as a result of the accelerated pace of global aging. As one of the most common malignant tumors of the head and neck, OSCC has seriously threatened human health and welfare due to its high recurrence and metastasis rate. Previous studies reported that factors such as TME, aberrant gene expression, and immune infiltration may be involved in the occurrence of tumors [32–34]. However, the molecular mechanism of OSCC pathogenesis has not yet been elucidated.

*IGF2BP2* is a member of the IGF2 mRNA-binding protein family. Many studies have shown that *IGF2BP2* is abnormally expressed in pancreatic cancer, liver cancer, thyroid cancer, and other malignant tumors [35]. Overexpression of *IGF2BP2* can promote tumor cell proliferation, stimulate migration and invasion, inhibit cell apoptosis, and accelerate tumor progression [36]. The study of Wang et al. showed that *IGF2BP2* up-regulated the expression of *circ 0000745* through *microRNA-3187-3p/ErbB4/PI3K/Akt* axis and promoted the aggressiveness and stemness of ovarian cancer cells [37]. Deng et al. confirmed that *IGF2PB2* can specifically bind to *TP53I11, PKP2, BMP6, CFH* and *COL1A1*, through ECM–receptor interaction, cytokine–cytokine receptor interaction, and TGF-β signaling pathway in Alz play an important regulatory role in Haimer’s disease [38]. Other research indicates that *IGF2BP2* plays a regulatory role in the pathological mechanisms of Lung ischemia–reperfusion injury (LIRI), Hemoglobin H-Constant Spring disease (HbH-CS), Autoimmune inflammation, and other diseases [39–41]. So far, there are few studies on *IGF2BP2* in OSCC. The study of Chou et al. found that patients with oral cancer and the *IGF2BP2* rs11705701 GA+AA, rs4402960 GT+TT, and rs1470579 AC+CC genotypes had higher risk in terms of clinical stage, tumor size, and lymph node metastasis compared with those with the *IGF2BP2* rs11705701 GG, rs4402960 GG, and rs1470579 AA genotypes. Studies have confirmed that HOXB-AS3 encodes a protein that directly interacts with *IGF2BP2* and promotes the proliferation and viability of OSCC cell lines by stabilizing c-Myc [42]. This study confirmed that the expression of *IGF2BP2* was significantly increased in OSCC tumor tissues after combining TCGA and GEO datasets, Oncomine, and clinical samples. The results showed that there is a statistical correlation between *IGF2BP2* and the T stage, the clinical stage of OSCC. In addition, aberrant expression of *IGF2BP2* is significantly associated with poor prognosis and overall survival rate in OSCC. Collectively, these results suggest that *IGF2BP2* may act as an oncogene to promote the occurrence of OSCC, and hopefully become a potential predictor of the prognosis of OSCC patients.

To explore the molecular function and potential mechanism of *IGF2BP2* in OSCC, samples were divided into *IGF2BP2-*high and *IGF2BP2-*low expression groups according to the expression of *IGF2BP2*, and further analysis was carried out using the GSEA software (version 4.1.0). For the C2 set defined by MSigDB, results showed that the immune and inflammation-related signaling pathways enriched in the *IGF2BP2*-high expression group include adipocytokine signaling pathway, insulin signaling pathway, and endocytosis, while tumorigenesis-related signaling pathways include Notch signaling pathway, P53 signaling pathway, WNT signaling pathway, ERBB signaling pathway, and phosphatidylinositol signaling pathway. Notably, adipocytokines is a type of soluble factor produced by adipose tissue, including adiponectin, leptin, resistin, and other components [43]. A previous study reported that the increase in leptin levels can significantly increase the expression of PD-1 and increase the exhaustion of CD8^+^ T cells in the TME, thereby affecting antitumor immunotherapy [44]. Endocytosis is an energy-dependent process that internalizes cell surface receptors through pinocytosis, phagocytosis, or receptor-mediated endocytosis, and is a very potential mechanism in regulating tumor metastasis. Many endocytic proteins are dysregulated in cancer and regulate tumor metastasis, especially migration and invasion [45]. Insulin is a cancer-related regulatory peptide. Studies have confirmed that IGF1R, one of the receptors associated with the insulin signaling pathway, can significantly promote proliferation of OSCC cells, and affect the occurrence and development of OSCC [46]. The Pi3k-akt signaling pathway, one of the phosphatidylinositol signaling systems, has been deeply studied in a variety of cancers. For example, results have shown that *IGF2BP2* can promote the progression of pancreatic cancer by activating this pathway [47]. On the other hand, various immune activities and metabolic functions, including apoptosis, glycolysis, pi3k-akt-mtor signaling, and mtorc1 signaling were enriched in the HALLMARK gene sets of the *IGF2BP2*-high expression group. It is worth noting that tumor cells favor glycolysis as the main source of energy metabolism due to the Warburg effect. One study reported that overexpression of *IGF2BP2* can promote glycolysis and stimulate tumor cell proliferation, thereby affecting the occurrence and development of tumors [48]. mTORC1 is one of the two complexes of mTOR (mammalian target of rapamycin), and is also a regulator of immune cell metabolism. Research has confirmed that *IGF2BP2* can regulate the cap-independent translation of IGF2 mRNA through dual phosphorylation with mTOR [49]. Collectively, these results indicate that *IGF2BP2* is involved in the tumor and immune-related KEGG pathway. The findings of the present study suggest that high expression of *IGF2BP2* can be used to predict poor prognosis and survival rate of OSCC patients. The reason may be that *IGF2BP2* modulates OSCC by affecting these signaling pathways, thereby resulting in poor prognosis for OSCC patients.

The results of the GEIPA and TIMER co-expression analyses indicated that *IGF2BP2* was correlated with *HMGA2, PHLDB2*, and *YEATS2*, which are involved in the inflammation/immune response or tumorigenesis [50–52]. In this study, results indicated that the expression of *IGF2BP2* co-expressed genes (*HMGA2*, *PHLDB2*, and *YEATS2*) was significantly increased in OSCC samples, and was correlated with a variety of TICs. Furthermore, CIBERSORT analysis based on the differential expression of *IGF2BP2* was used to evaluate the distribution ratio of TICs in OSCC. The TICs were then screened together through correlation and differential analyses. Results indicated that seven TICs were associated with *IGF2BP2* expression, seven TICs were associated with *HGMA2* expression, eight TICs were associated with *PHLDB2* expression, and five TICs were associated with *YEATS2* expression. These results suggest that *IGF2BP2* and its co-expressed genes may be involved in the immune response during the occurrence of OSCC, thereby leading to a poor prognosis in OSCC patients.

At present, most bioinformatics studies only focus on a gene in a single database, and there are relatively few model analysis of multidatabase joint gene prediction. In addition, due to the limited sample size of a single dataset, the results of differential gene analysis may be biased, resulting in no biological effects. Compared with other single dataset analysis, the present study combined oncomine database, multiple datasets of GEO (GSE31056, GSE42743, GSE51010) and TCGA database for gene prediction model analysis to identify possible biomarkers in OSCC, which laid a more reliable and accurate foundation for our research. At the same time, we combined these data with patient data in our hospital to verify the existence of biomarkers.

Nevertheless, our research still has some limitations: (1) our research is verified on the basis of database data analysis combined with our own clinical samples, but the relatively small clinical sample size is still the limitation of the present study. (2) Bioinformatics analysis is only a prediction tool based on public database. Its operation process is cumbersome, involving the setting and adjustment of a variety of software analysis parameters. A certain technical threshold is required to ensure the accuracy of prediction results. (3) The impact of specific characteristics such as race, smoking history, drinking history and HPV history on prognosis was not analyzed in detail. Therefore, these will also be the focus of our next research.

## Conclusion

In summary, the present study has confirmed the high expression of *IGF2BP2* in OSCC, the survival and prognosis of patients in the *IGF2BP2*-high expression group is poor, and that the high expression of *IGF2BP2* is associated with some clinicopathological parameters such as T stage and clinical stage. In addition, the study found that *IGF2BP2* and its co-expressed genes (*HMGA2, PHLDB2*, and *YEATS2*) are all associated with a variety of TICs in OSCC tumor samples. Therefore, the present study has revealed the potential role of *IGF2BP2* in tumor immunology and its prognostic value. It is evident that *IGF2BP2* has the potential to be a prognostic biomarker and therapeutic target for OSCC. However, further studies should be conducted to elucidate the specific mechanism of the interaction of *IGF2BP2* and its co-expressed genes with TICs.

## Supplementary Material

Supplementary Figures S1-S2 and Tables S1-S6

## Data Availability

The datasets generated and analyzed in the present study are available in the TCGA database (https://portal.gdc.cancer.gov) and the NCBI’s GEO (https://www.ncbi.nlm.nih.gov/geo/).

## Abbreviations

BP: Biological process
CC: Cellular component
COAD: colorectal adenocarcinoma
DC: dendritic cell
DEG: differentially expressed gene
FDR: false discovery rate
GEIPA: Gene expression profiling interactive analysis
GEO: Gene Expression Omnibus
GO: gene ontology
GSEA: gene set enrichment analysis
HNSC: head and neck squamous cell carcinoma
HPV: human papilloma virus
*IGF2BP2*: insulin-like growth factor 2 mRNA-binding protein 2
KEGG: Kyoto Encyclopedia of Genes and Genomes
KS: Kolmogorov-Smirnov test
MF: Molecular function
MSigDB: Molecular Signatures Database
mTOR: mammalian target of rapamycin
NCBI: National Center for Biotechnology Information
NOM: nominal
OSCC: oral squamous cell carcinoma
TCGA: The Cancer Genome Atlas
TIC: tumor-infiltrating immune cell
TIMER: Tumor Immune estimation resource
TME: tumor microenvironment
Treg: Regulatory T cells
