# Supplementary material for: IGF2BP2 maybe a novel prognostic biomarker in oral squamous cell carcinoma
Source: Biosci Rep. 2022 Feb 18;42(2):BSR20212119. doi: 10.1042/BSR20212119 (PMC8859425; doi:10.1042/BSR20212119)
Supplement: Supplementary Figures S1-S2 and Tables S1-S6 [file BSR-2021-2119_supp.pdf]

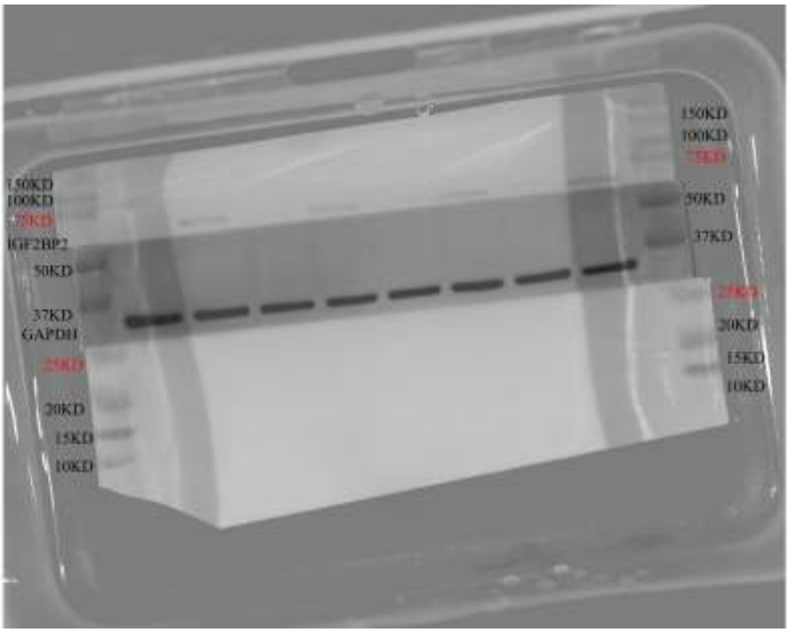

Supplementary Fig. 1 Western blot experiment results of tissue samples from OSCC patients.

This figure shows the Western blot experiment results of OSCC tumor samples and non-tumor samples of sample 1 to sample 4.

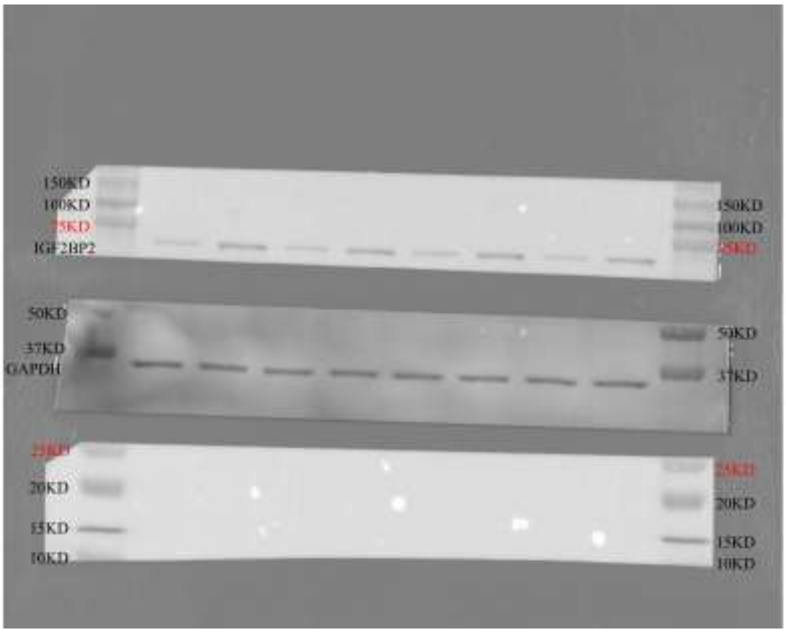

Supplementary Fig. 2 Western blot experiment results of tissue samples from OSCC patients.

This figure shows the Western blot experiment results of OSCC tumor samples and non-tumor samples of sample 5 to sample 8.

Supplementary table 1 The RNA-Seq gene expression data of IGF2BP2 in 341 OSCC samples of TCGA database. This table shows the expression matrix of IGF2BP2 in 341 OSCC samples from TCGA database used in this study.

ID	IGF2BP2	Type
TCGA-CV-7416-11A-01R-2081-07	4.838016	Normal
TCGA-CV-6960-11A-01R-2016-07	8.029173	Normal
TCGA-HD-8635-11A-01R-2403-07	0.62889	Normal
TCGA-CV-6939-11A-01R-1915-07	1.608976	Normal
TCGA-H7-A6C4-11A-21R-A466-07	0.335578	Normal
TCGA-CV-7434-11A-01R-2132-07	3.22101	Normal
TCGA-CV-7425-11A-01R-2081-07	9.598407	Normal
TCGA-CV-7255-11A-01R-2016-07	5.98311	Normal
TCGA-CV-7235-11A-01R-2016-07	5.294149	Normal
TCGA-CV-6933-11A-01R-1915-07	2.074432	Normal
TCGA-HD-A6HZ-11A-11R-A31N-07	0.558399	Normal
TCGA-CV-7103-11A-01R-2016-07	1.875922	Normal
TCGA-CV-7097-11A-01R-2016-07	2.081183	Normal
TCGA-CV-7178-11A-01R-2016-07	1.88119	Normal
TCGA-WA-A7GZ-11A-11R-A34R-07	0.576195	Normal
TCGA-CV-6959-11A-01R-1915-07	0.732115	Normal
TCGA-CV-7183-11A-01R-2016-07	0.100496	Normal
TCGA-CV-7438-11A-01R-2132-07	2.660679	Normal
TCGA-CV-7423-11A-01R-2081-07	0.453654	Normal
TCGA-CV-6936-11A-01R-1915-07	0.485891	Normal
TCGA-CV-6956-11A-01R-2016-07	0.328126	Normal
TCGA-CV-7091-11A-01R-2016-07	1.433702	Normal
TCGA-CV-7432-11A-01R-2132-07	5.731585	Normal
TCGA-CV-7238-11A-01R-2016-07	1.744324	Normal
TCGA-CV-6934-11A-01R-1915-07	9.112463	Normal
TCGA-CV-6961-11A-01R-1915-07	3.361487	Normal
TCGA-CV-6938-11A-01R-1915-07	2.267315	Normal
TCGA-CV-7252-11A-01R-2016-07	0.611739	Normal
TCGA-HD-A6I0-11A-11R-A31N-07	1.746857	Normal
TCGA-CV-6955-11A-01R-2016-07	1.685435	Normal
TCGA-CV-7414-01A-11R-2081-07	1.818111	Tumor
TCGA-CQ-6227-01A-11R-1915-07	30.85148	Tumor
TCGA-CV-7432-01A-11R-2132-07	27.94551	Tumor
TCGA-UF-A7JD-01A-11R-A34R-07	29.78326	Tumor
TCGA-CQ-6225-01A-11R-1915-07	53.70028	Tumor
TCGA-BA-4074-01A-01R-1436-07	5.728246	Tumor

TCGA-CQ-5324-01A-01R-1686-07	19.39155	Tumor
TCGA-BB-A5HU-01A-11R-A28V-07	13.3042	Tumor
TCGA-CQ-6228-01A-11R-1915-07	28.6124	Tumor
TCGA-CN-5367-01A-01R-1436-07	13.69675	Tumor
TCGA-CV-5971-01A-11R-1686-07	5.289856	Tumor
TCGA-CN-6011-01A-11R-1686-07	21.97387	Tumor
TCGA-CN-6019-01A-11R-1686-07	11.67105	Tumor
TCGA-BA-4077-01B-01R-1436-07	28.66238	Tumor
TCGA-CX-7219-01A-11R-2016-07	14.42622	Tumor
TCGA-UF-A71A-06A-11R-A39I-07	34.51363	Tumor
TCGA-CQ-7068-01A-11R-2081-07	25.24785	Tumor
TCGA-CV-7427-01A-11R-2081-07	19.56598	Tumor
TCGA-P3-A6T0-01A-12R-A34R-07	10.35033	Tumor
TCGA-CQ-A4C6-01A-11R-A24Z-07	18.16148	Tumor
TCGA-P3-A5QA-01A-11R-A28V-07	20.48041	Tumor
TCGA-CQ-6218-01A-11R-1915-07	7.721948	Tumor
TCGA-CR-6488-01A-12R-2081-07	19.27947	Tumor
TCGA-CN-5370-01A-01R-2016-07	18.08488	Tumor
TCGA-IQ-A61J-01A-11R-A30B-07	12.93648	Tumor
TCGA-CQ-5325-01A-01R-1686-07	8.757355	Tumor
TCGA-DQ-5624-01A-01R-1873-07	23.91103	Tumor
TCGA-CQ-6224-01A-11R-1915-07	14.19381	Tumor
TCGA-BA-5558-01A-01R-1514-07	31.35936	Tumor
TCGA-H7-8501-01A-11R-2403-07	26.03806	Tumor
TCGA-CV-6939-01A-11R-1915-07	3.764882	Tumor
TCGA-CV-7425-01A-11R-2081-07	11.84039	Tumor
TCGA-HL-7533-01A-11R-2232-07	13.17781	Tumor
TCGA-CQ-A4CB-01A-11R-A24Z-07	61.77949	Tumor
TCGA-QK-A64Z-01A-11R-A30B-07	6.924213	Tumor
TCGA-CR-7368-01A-11R-2132-07	1.152206	Tumor
TCGA-CV-A6JE-01A-11R-A31N-07	4.627556	Tumor
TCGA-DQ-5631-01A-01R-1873-07	30.6699	Tumor
TCGA-QK-A6IG-01A-11R-A31N-07	15.45677	Tumor
TCGA-CQ-7063-01A-11R-2403-07	19.35861	Tumor
TCGA-CR-6492-01A-12R-2081-07	28.36023	Tumor
TCGA-P3-A6T2-01A-11R-A34R-07	25.85297	Tumor
TCGA-UP-A6WW-01A-12R-A34R-07	0.354457	Tumor
TCGA-CV-A6JZ-01A-11R-A31N-07	44.76815	Tumor
TCGA-CV-A6JY-01A-11R-A31N-07	4.791406	Tumor
TCGA-IQ-A6SH-01A-12R-A34R-07	9.526024	Tumor
TCGA-CV-A45O-01A-21R-A24H-07	7.394123	Tumor
TCGA-BA-A6DE-01A-22R-A31N-07	20.27738	Tumor
TCGA-CX-7086-01A-11R-2081-07	17.78672	Tumor
TCGA-CR-7379-01A-11R-2016-07	14.46432	Tumor

TCGA-D6-A6EM-01A-21R-A31N-07	19.62555	Tumor
TCGA-CV-6433-01A-11R-1686-07	4.325004	Tumor
TCGA-CQ-A4CI-01A-11R-A266-07	27.44826	Tumor
TCGA-T2-A6WX-01A-12R-A34R-07	17.31597	Tumor
TCGA-CQ-A4CA-01A-11R-A24Z-07	5.748949	Tumor
TCGA-D6-6823-01A-11R-1915-07	41.05676	Tumor
TCGA-CR-6491-01A-11R-1873-07	17.88746	Tumor
TCGA-CV-A6JU-01A-11R-A31N-07	7.113439	Tumor
TCGA-CV-A45X-01A-21R-A24Z-07	14.6673	Tumor
TCGA-CV-7407-01A-11R-2081-07	12.51083	Tumor
TCGA-IQ-A61H-01A-11R-A30B-07	9.114745	Tumor
TCGA-CV-6951-01A-11R-1915-07	28.48342	Tumor
TCGA-CN-4733-01A-02R-1873-07	7.930819	Tumor
TCGA-CV-7263-01A-11R-2016-07	14.83473	Tumor
TCGA-F7-A61W-01A-11R-A28V-07	22.71932	Tumor
TCGA-QK-A8Z9-01B-11R-A39I-07	7.298535	Tumor
TCGA-BB-A6UO-01A-12R-A34R-07	10.45049	Tumor
TCGA-BA-5556-01A-01R-1514-07	18.30113	Tumor
TCGA-CQ-5327-01A-01R-1686-07	20.09552	Tumor
TCGA-CR-7393-01A-11R-2016-07	3.741296	Tumor
TCGA-CV-7090-01A-11R-2016-07	6.399096	Tumor
TCGA-QK-A6IH-01A-11R-A31N-07	4.001046	Tumor
TCGA-F7-A624-01A-22R-A30B-07	15.70343	Tumor
TCGA-CV-7100-01A-11R-2016-07	61.10415	Tumor
TCGA-CR-6471-01A-11R-1873-07	6.075484	Tumor
TCGA-CV-7235-01A-11R-2016-07	6.545092	Tumor
TCGA-CN-6994-01A-11R-1915-07	19.64885	Tumor
TCGA-CQ-6223-01A-11R-1915-07	14.52344	Tumor
TCGA-CN-4725-01A-01R-1436-07	16.10303	Tumor
TCGA-C9-A47Z-01A-11R-A24H-07	37.11661	Tumor
TCGA-CN-6017-01A-11R-1686-07	10.24288	Tumor
TCGA-CV-6945-01A-11R-1915-07	15.28855	Tumor
TCGA-CX-A4AQ-01A-11R-A24Z-07	5.919378	Tumor
TCGA-CQ-6229-01A-11R-1915-07	19.05968	Tumor
TCGA-CN-5358-01A-01R-1514-07	8.058786	Tumor
TCGA-BA-6872-01A-11R-1873-07	11.56215	Tumor
TCGA-QK-A652-01A-11R-A30B-07	9.160594	Tumor
TCGA-CV-A6JO-01B-11R-A34R-07	19.29073	Tumor
TCGA-CV-A45P-01A-11R-A24H-07	11.49124	Tumor
TCGA-CV-6937-01A-11R-2016-07	13.77865	Tumor
TCGA-CN-6018-01A-11R-1686-07	24.54597	Tumor
TCGA-CV-5977-01A-11R-1686-07	21.45865	Tumor
TCGA-CV-7568-01A-11R-2232-07	4.820813	Tumor
TCGA-P3-A6T6-01A-11R-A34R-07	7.926477	Tumor

TCGA-CV-5436-01A-01R-1514-07	11.76315	Tumor
TCGA-MT-A67A-01A-11R-A30B-07	2.780989	Tumor
TCGA-CV-5966-01A-11R-1686-07	36.43607	Tumor
TCGA-CN-6016-01A-11R-1686-07	13.34788	Tumor
TCGA-CN-6024-01A-11R-1686-07	8.896562	Tumor
TCGA-CR-7401-01A-11R-2016-07	7.690861	Tumor
TCGA-DQ-7592-01A-11R-2081-07	44.83367	Tumor
TCGA-4P-AA8J-01A-11R-A39I-07	9.548802	Tumor
TCGA-UF-A71E-01A-31R-A34R-07	23.57489	Tumor
TCGA-CV-7253-01A-11R-2016-07	44.1252	Tumor
TCGA-CN-4730-01A-01R-1436-07	35.50191	Tumor
TCGA-CV-6952-01A-11R-1915-07	27.80131	Tumor
TCGA-CQ-6220-01A-11R-1915-07	30.50702	Tumor
TCGA-CV-7097-01A-11R-2016-07	25.41434	Tumor
TCGA-BA-7269-01A-11R-2016-07	8.606186	Tumor
TCGA-CN-4741-01A-01R-1436-07	8.894835	Tumor
TCGA-CV-6959-01A-11R-1915-07	43.9221	Tumor
TCGA-CQ-7065-01A-11R-2081-07	17.30421	Tumor
TCGA-CV-5442-01A-01R-1514-07	11.80151	Tumor
TCGA-CX-7082-01A-11R-2016-07	41.76764	Tumor
TCGA-D6-A6EN-01A-11R-A31N-07	16.76241	Tumor
TCGA-CN-4740-01A-01R-1436-07	3.116214	Tumor
TCGA-QK-A6IJ-01A-11R-A31N-07	10.83674	Tumor
TCGA-BA-A6DB-01A-11R-A30B-07	4.23568	Tumor
TCGA-CR-7397-01A-11R-2016-07	7.904125	Tumor
TCGA-CQ-5330-01A-01R-1686-07	18.33266	Tumor
TCGA-CR-7369-01A-11R-2132-07	14.62829	Tumor
TCGA-CV-5970-01A-11R-1686-07	11.23604	Tumor
TCGA-CV-7103-01A-21R-2016-07	37.7197	Tumor
TCGA-D6-8569-01A-11R-2403-07	37.18478	Tumor
TCGA-CV-6938-01A-11R-1915-07	21.63656	Tumor
TCGA-CQ-5334-01A-01R-1686-07	20.44849	Tumor
TCGA-IQ-A61E-01A-22R-A30B-07	31.02575	Tumor
TCGA-HD-8635-01A-11R-2403-07	22.50001	Tumor
TCGA-CR-7391-01A-11R-2016-07	6.62793	Tumor
TCGA-CN-4729-01A-01R-1436-07	14.33241	Tumor
TCGA-CQ-5329-01A-01R-1686-07	30.35836	Tumor
TCGA-CV-7411-01A-11R-2081-07	7.733621	Tumor
TCGA-CQ-6221-01A-11R-2081-07	8.482994	Tumor
TCGA-DQ-5630-01A-01R-1873-07	8.57258	Tumor
TCGA-BA-A6DD-01A-12R-A31N-07	16.3261	Tumor
TCGA-CV-6934-01A-11R-1915-07	13.09847	Tumor
TCGA-CV-7099-01A-41R-2016-07	9.261619	Tumor
TCGA-CV-A45U-01A-12R-A24H-07	29.37404	Tumor

TCGA-H7-7774-01A-21R-2081-07	4.293976	Tumor
TCGA-CV-7435-01A-11R-2132-07	30.5083	Tumor
TCGA-CN-6020-01A-11R-1686-07	11.16907	Tumor
TCGA-P3-A6T3-01A-11R-A34R-07	17.07136	Tumor
TCGA-C9-A480-01A-12R-A24H-07	38.22905	Tumor
TCGA-CQ-A4CE-01A-11R-A266-07	7.284683	Tumor
TCGA-CV-6961-01A-21R-1915-07	19.73882	Tumor
TCGA-CV-A6JN-01A-11R-A31N-07	5.327183	Tumor
TCGA-CV-7438-01A-21R-2132-07	27.76909	Tumor
TCGA-H7-8502-01A-11R-2403-07	59.46221	Tumor
TCGA-CN-6998-01A-23R-2016-07	39.73718	Tumor
TCGA-P3-A6T7-01A-11R-A34R-07	28.98883	Tumor
TCGA-CR-6493-01A-11R-1873-07	29.67996	Tumor
TCGA-WA-A7H4-01A-21R-A34R-07	9.043073	Tumor
TCGA-UF-A71A-01A-22R-A34R-07	25.28	Tumor
TCGA-CV-7180-01A-11R-2016-07	16.14871	Tumor
TCGA-CV-A465-01A-11R-A266-07	22.81529	Tumor
TCGA-CV-7446-01A-11R-2232-07	46.42513	Tumor
TCGA-HD-7831-01A-11R-2132-07	16.51814	Tumor
TCGA-BB-8601-01A-11R-2403-07	15.60339	Tumor
TCGA-CV-5979-01A-11R-1686-07	20.52762	Tumor
TCGA-CR-7392-01A-11R-2016-07	16.50869	Tumor
TCGA-DQ-5625-01A-01R-1873-07	3.873753	Tumor
TCGA-CV-5973-01A-11R-1686-07	33.91398	Tumor
TCGA-CV-6933-01A-11R-1915-07	20.51769	Tumor
TCGA-CV-7236-01A-11R-2016-07	23.35882	Tumor
TCGA-CV-6953-01A-11R-1915-07	9.102598	Tumor
TCGA-CN-5364-01A-01R-1436-07	20.53391	Tumor
TCGA-HD-A633-01A-11R-A28V-07	39.84728	Tumor
TCGA-CN-4728-01A-01R-1436-07	15.01338	Tumor
TCGA-CV-A6K2-01A-11R-A31N-07	11.8777	Tumor
TCGA-CR-7390-01A-11R-2016-07	59.26749	Tumor
TCGA-RS-A6TO-01A-32R-A34R-07	29.58158	Tumor
TCGA-CN-A642-01A-12R-A30B-07	18.03591	Tumor
TCGA-CV-7252-01A-11R-2016-07	24.86138	Tumor
TCGA-CV-A45R-01A-11R-A24H-07	8.57595	Tumor
TCGA-CV-A463-01A-11R-A266-07	8.931775	Tumor
TCGA-HD-7832-01A-11R-2132-07	21.38517	Tumor
TCGA-CV-6936-01A-11R-1915-07	5.355899	Tumor
TCGA-H7-A6C4-01A-11R-A30B-07	10.32002	Tumor
TCGA-CV-7091-01A-11R-2016-07	46.73252	Tumor
TCGA-UF-A71B-01A-12R-A34R-07	8.964827	Tumor
TCGA-D6-6515-01A-21R-1873-07	7.473991	Tumor
TCGA-CV-7254-01A-11R-2016-07	11.43372	Tumor

TCGA-CV-7429-01A-11R-2132-07	19.72163	Tumor
TCGA-CV-5976-01A-11R-1686-07	18.14054	Tumor
TCGA-BA-5151-01A-01R-1436-07	20.42175	Tumor
TCGA-CN-A63V-01A-11R-A466-07	10.03396	Tumor
TCGA-QK-A8Z7-01A-11R-A39I-07	77.74676	Tumor
TCGA-CV-7178-01A-21R-2016-07	2.349006	Tumor
TCGA-UF-A7JC-01A-21R-A34R-07	14.53556	Tumor
TCGA-CV-7255-01A-11R-2016-07	22.28357	Tumor
TCGA-P3-A6T8-01A-11R-A34R-07	39.96449	Tumor
TCGA-CX-7085-01A-21R-2016-07	10.62148	Tumor
TCGA-KU-A66T-01A-11R-A30B-07	18.84359	Tumor
TCGA-D6-A6EO-01A-11R-A31N-07	29.92763	Tumor
TCGA-MT-A7BN-01A-12R-A34R-07	2.632273	Tumor
TCGA-UF-A719-01A-12R-A34R-07	20.58342	Tumor
TCGA-WA-A7GZ-01A-11R-A34R-07	19.9766	Tumor
TCGA-BA-4075-01A-01R-1436-07	15.54537	Tumor
TCGA-KU-A6H8-01A-21R-A34R-07	0.715671	Tumor
TCGA-CV-7413-01A-11R-2081-07	23.71067	Tumor
TCGA-CQ-5326-01A-01R-1873-07	23.3592	Tumor
TCGA-BA-A6D8-01A-31R-A31N-07	10.31247	Tumor
TCGA-F7-A50J-01A-21R-A28V-07	19.75512	Tumor
TCGA-HD-A6I0-01A-11R-A31N-07	5.681175	Tumor
TCGA-CV-7416-01A-11R-2081-07	13.09393	Tumor
TCGA-CQ-5331-01A-02R-1873-07	11.50032	Tumor
TCGA-CV-A45T-01A-11R-A24H-07	5.651407	Tumor
TCGA-CN-5369-01A-01R-1436-07	15.87002	Tumor
TCGA-CR-7376-01A-11R-2132-07	10.36029	Tumor
TCGA-CN-5373-01A-01R-1436-07	13.77579	Tumor
TCGA-F7-A61S-01A-11R-A28V-07	18.45225	Tumor
TCGA-P3-A6T4-01A-11R-A34R-07	18.44316	Tumor
TCGA-D6-6516-01A-11R-1873-07	6.439845	Tumor
TCGA-CQ-7071-01A-12R-A30B-07	14.49626	Tumor
TCGA-IQ-7632-01A-11R-2081-07	13.71637	Tumor
TCGA-CV-A6JT-01A-11R-A31N-07	7.098611	Tumor
TCGA-CV-6941-01A-11R-1915-07	16.47071	Tumor
TCGA-UF-A7JA-01A-12R-A34R-07	17.65815	Tumor
TCGA-P3-A6T5-01A-11R-A34R-07	3.130358	Tumor
TCGA-CN-6013-01A-11R-1686-07	18.90971	Tumor
TCGA-CN-4734-01A-01R-1436-07	12.44199	Tumor
TCGA-CQ-5323-01A-01R-1686-07	13.34905	Tumor
TCGA-CR-7380-01A-11R-2016-07	9.590946	Tumor
TCGA-DQ-7588-01A-11R-2081-07	25.63876	Tumor
TCGA-CQ-7069-01A-11R-2403-07	24.11146	Tumor
TCGA-CV-A6K0-01B-21R-A31N-07	8.577369	Tumor

TCGA-CR-7373-01A-11R-2016-07	24.12001	Tumor
TCGA-D6-A4Z9-01A-11R-A24Z-07	15.80998	Tumor
TCGA-D6-6825-01A-21R-1915-07	21.34184	Tumor
TCGA-CQ-A4C9-01A-11R-A24Z-07	18.49954	Tumor
TCGA-CN-6995-01A-31R-2016-07	57.818	Tumor
TCGA-CQ-A4CG-01A-11R-A266-07	22.76223	Tumor
TCGA-CQ-A4CD-01A-21R-A24Z-07	13.00679	Tumor
TCGA-CV-6003-01A-11R-1686-07	27.82443	Tumor
TCGA-IQ-A61G-01A-11R-A30B-07	23.80132	Tumor
TCGA-CN-4736-01A-01R-1436-07	10.96505	Tumor
TCGA-CQ-6222-01A-11R-1915-07	4.580626	Tumor
TCGA-P3-A5QF-01A-11R-A28V-07	13.7303	Tumor
TCGA-T3-A92N-01A-11R-A39I-07	41.54612	Tumor
TCGA-HD-A4C1-01A-11R-A24H-07	19.55443	Tumor
TCGA-CV-6441-01A-11R-1686-07	13.96697	Tumor
TCGA-CR-7386-01A-11R-2016-07	12.25557	Tumor
TCGA-CN-4726-01A-01R-1436-07	11.73919	Tumor
TCGA-CN-4742-01A-02R-1514-07	12.75374	Tumor
TCGA-IQ-A6SG-01A-12R-A34R-07	18.12935	Tumor
TCGA-CN-4737-01A-01R-1436-07	18.6675	Tumor
TCGA-CN-A49A-01A-11R-A24H-07	33.91156	Tumor
TCGA-F7-8489-01A-31R-2403-07	11.03058	Tumor
TCGA-CN-A498-01A-11R-A24H-07	4.960546	Tumor
TCGA-HD-8634-01A-11R-2403-07	11.59003	Tumor
TCGA-CV-A6JD-01A-11R-A31N-07	15.49452	Tumor
TCGA-CN-4731-01A-01R-1436-07	23.75699	Tumor
TCGA-CV-7423-01A-11R-2081-07	13.21792	Tumor
TCGA-CN-5359-01A-01R-1436-07	11.95947	Tumor
TCGA-BB-A5HZ-01A-21R-A28V-07	15.12189	Tumor
TCGA-CR-7395-01A-11R-2016-07	11.93937	Tumor
TCGA-IQ-7631-01A-11R-2081-07	1.382554	Tumor
TCGA-CR-7367-01A-11R-2016-07	10.02137	Tumor
TCGA-HD-A6HZ-01A-12R-A31N-07	17.49711	Tumor
TCGA-CQ-A4C7-01A-11R-A24Z-07	31.88819	Tumor
TCGA-CV-A45Q-01A-11R-A24H-07	5.22751	Tumor
TCGA-UF-A7JT-01A-11R-A34R-07	18.44465	Tumor
TCGA-CR-6484-01A-11R-1873-07	5.687758	Tumor
TCGA-CR-7382-01A-11R-2132-07	16.92979	Tumor
TCGA-CV-7238-01A-11R-2016-07	5.662064	Tumor
TCGA-BA-A6DJ-01A-11R-A30B-07	16.46969	Tumor
TCGA-F7-A50G-01A-11R-A266-07	12.63978	Tumor
TCGA-CV-6940-01A-11R-1915-07	12.61507	Tumor
TCGA-CQ-5332-01A-01R-1686-07	28.59974	Tumor
TCGA-MT-A51X-01A-11R-A266-07	9.47113	Tumor

TCGA-CV-6954-01A-11R-1915-07	33.44162	Tumor
TCGA-CQ-A4CH-01A-11R-A266-07	30.84969	Tumor
TCGA-CV-A45V-01A-21R-A24Z-07	4.32669	Tumor
TCGA-CV-6956-01A-21R-2016-07	28.9945	Tumor
TCGA-CQ-5333-01A-01R-2403-07	20.07054	Tumor
TCGA-CQ-7072-01A-21R-A30B-07	40.53841	Tumor
TCGA-CV-7183-01A-11R-2016-07	9.643647	Tumor
TCGA-QK-A6VB-01A-12R-A34R-07	8.897756	Tumor
TCGA-CV-A464-01A-11R-A266-07	1.534557	Tumor
TCGA-CV-6942-01A-21R-2016-07	8.012246	Tumor
TCGA-CV-6955-01A-11R-2016-07	0.543831	Tumor
TCGA-T2-A6X2-01A-12R-A34R-07	13.25664	Tumor
TCGA-CN-6996-01A-11R-1915-07	16.45306	Tumor
TCGA-CV-7428-01A-11R-2132-07	21.29637	Tumor
TCGA-BA-A6DG-01A-21R-A30B-07	24.07745	Tumor
TCGA-CV-A6JM-01A-11R-A31N-07	16.39556	Tumor
TCGA-QK-A6II-01A-11R-A31N-07	8.909452	Tumor
TCGA-BA-6873-01A-11R-1873-07	14.64808	Tumor
TCGA-MT-A67F-01A-11R-A30B-07	14.59282	Tumor
TCGA-CV-7102-01A-11R-2016-07	24.3212	Tumor
TCGA-BA-5557-01A-01R-1514-07	24.59781	Tumor
TCGA-CR-7365-01A-11R-2016-07	32.11855	Tumor
TCGA-MT-A67D-01A-31R-A30B-07	12.81283	Tumor
TCGA-D6-6827-01A-11R-1915-07	7.745325	Tumor
TCGA-CV-7434-01A-11R-2132-07	20.24224	Tumor
TCGA-CV-6960-01A-41R-2016-07	32.40212	Tumor
TCGA-BA-5152-01A-02R-1873-07	5.555268	Tumor
TCGA-CR-7377-01A-11R-2016-07	7.149058	Tumor
TCGA-CV-6436-01A-11R-1686-07	8.326412	Tumor
TCGA-CR-7372-01A-11R-2016-07	7.475007	Tumor
TCGA-CV-6948-01A-11R-1915-07	27.79333	Tumor
TCGA-CV-7095-01A-21R-2016-07	13.8674	Tumor
TCGA-D6-A4ZB-01A-11R-A24Z-07	10.62158	Tumor
TCGA-CR-7394-01A-11R-2016-07	11.89523	Tumor
TCGA-CV-7104-01A-11R-2016-07	17.62246	Tumor
TCGA-UF-A7JO-01A-11R-A34R-07	10.94287	Tumor
TCGA-QK-AA3K-01A-11R-A39I-07	7.835285	Tumor
TCGA-BB-4224-01A-01R-1436-07	9.364302	Tumor
TCGA-CV-A468-01A-11R-A266-07	3.695313	Tumor
TCGA-UF-A7JS-01A-11R-A34R-07	7.867345	Tumor
TCGA-CQ-6219-01A-11R-1915-07	17.87503	Tumor

Supplementary table 2 The clinical data of OSCC samples in TCGA database. This table shows the clinical information of OSCC samples from TCGA database used in this study.

Id	futime	fustat	age	gender	grade	stage	T	M	N
TCGA-CV-6433	641	0	57	MALE	G3	Stage II	T2	unknow	N0
TCGA-BA-A6DE	440	0	70	FEMALE	G2	Stage II	T2	M0	N0
TCGA-MT-A51X	242	0	30	MALE	G1	Stage IVA	T1	M0	N2b
TCGA-MT-A7BN	469	0	74	MALE	G3	Stage IVA	unknow	unknow	unknow
TCGA-D6-A4Z9	539	0	59	MALE	G2	Stage IVA	T2	M0	N2
TCGA-CR-6471	1202	1	58	MALE	G2	Stage IVA	T4a	M0	N1
TCGA-BA-5556	725	0	58	FEMALE	G3	Stage II	T2	unknow	N0
TCGA-CR-7379	1036	0	78	FEMALE	G2	Stage IVA	T4a	M0	N2b
TCGA-P3-A6T5	882	1	79	FEMALE	G2	Stage IVA	T4a	MX	N0
TCGA-CV-7425	1718	1	77	FEMALE	G1	Stage III	T3	unknow	NX
TCGA-CN-4725	1157	0	60	MALE	G2	Stage II	T2	unknow	N0
TCGA-CQ-7069	1274	0	77	FEMALE	G2	Stage II	T2	M0	N0
TCGA-CV-5971	701	0	60	MALE	G2	Stage IVA	T4a	unknow	N2a
TCGA-QK-A6IH	653	0	65	FEMALE	G2	Stage IVB	T4b	M0	N2b
TCGA-CV-A45T	4856	1	64	FEMALE	G3	unknow	T1	M0	N0
TCGA-CR-7401	1077	0	64	MALE	G2	Stage I	T1	M0	N0
TCGA-HD-8634	385	1	51	FEMALE	G2	Stage I	T1	MX	N0
TCGA-CQ-A4CB	893	0	59	MALE	G2	Stage III	T1	M0	N1
TCGA-CV-7255	64	1	32	FEMALE	G2	Stage IVA	T4a	unknow	N0
TCGA-CN-6995	112	1	78	MALE	G2	Stage IVA	T4a	unknow	N0
TCGA-CV-7435	4680	1	57	FEMALE	G2	Stage IVA	T3	unknow	N2b
TCGA-CN-6996	530	1	58	FEMALE	G2	Stage IVA	T3	unknow	N2b
TCGA-CQ-A4CE	897	0	76	FEMALE	G2	Stage II	T2	M0	N0
TCGA-CV-7416	763	1	29	FEMALE	GX	Stage IVA	T4a	unknow	unknow
TCGA-QK-A6II	284	1	52	MALE	G2	Stage IVA	T4a	M0	N2c
TCGA-CN-4730	817	0	62	MALE	G2	Stage IVA	T4a	unknow	N1
TCGA-BA-5558	1995	0	65	MALE	G1	unknow	TX	unknow	NX
TCGA-CV-7432	2570	1	79	MALE	G1	Stage II	T2	unknow	N0
TCGA-CR-7390	1508	0	67	MALE	G2	Stage IVA	T4a	M0	N0
TCGA-CQ-A4CI	950	0	73	MALE	G3	Stage III	T2	M0	N1
TCGA-CX-7086	573	0	53	MALE	G2	Stage III	T3	unknow	N1
TCGA-CV-5966	545	1	63	FEMALE	G3	Stage IVA	T4a	unknow	N1
TCGA-CN-4733	1586	0	61	MALE	G3	Stage III	T1	unknow	N1
TCGA-CV-A6JU	110	0	61	FEMALE	G2	Stage IVB	T4b	M0	N0
TCGA-CV-7236	144	1	77	FEMALE	G3	Stage IVA	T3	unknow	N2c
TCGA-CV-7568	927	1	48	FEMALE	GX	Stage IVA	T4a	unknow	NX
TCGA-IQ-A61K	161	1	70	FEMALE	G2	Stage IVA	T3	M0	N2b
TCGA-CV-A45U	1079	1	59	MALE	G1	Stage IVA	T4	M0	N2b
TCGA-CV-7183	3981	0	53	MALE	G1	Stage II	T2	unknow	N0

TCGA-QK-A6VB	641	0	66	MALE	G2	Stage IVA	T4a	M0	N0
TCGA-WA-A7H4	443	0	69	MALE	G3	Stage II	T2	unknow	N0
TCGA-CQ-6223	1428	0	69	MALE	G2	Stage II	T2	unknow	N0
TCGA-BB-7863	1025	0	43	FEMALE	G2	Stage III	T3	unknow	N0
TCGA-4P-AA8J	102	0	66	MALE	G2	Stage IVA	T2	MX	N2c
TCGA-UF-A7JS	680	1	59	MALE	G2	Stage IVA	T4a	M0	N2b
TCGA-DQ-5630	1030	0	73	MALE	G2	unknow	TX	unknow	NX
TCGA-D6-6516	773	0	69	MALE	G2	Stage I	T1	unknow	N0
TCGA-CV-5976	1478	0	50	MALE	G2	Stage IVA	T4a	unknow	N2b
TCGA-CQ-7064	1973	0	83	FEMALE	G2	Stage III	T3	M0	N1
TCGA-CX-7085	321	0	77	FEMALE	G2	Stage I	T1	unknow	N0
TCGA-CQ-7067	509	0	75	FEMALE	G3	Stage I	T1	unknow	N0
TCGA-CR-7369	1090	1	59	MALE	G2	Stage IVA	T4a	M0	N2b
TCGA-D6-6823	701	0	50	MALE	G2	Stage II	T2	unknow	N0
TCGA-CV-5436	584	1	65	MALE	G2	Stage IVA	T3	unknow	N2b
TCGA-CV-6948	1289	1	79	FEMALE	G2	Stage IVB	T4a	unknow	N3
TCGA-CQ-7068	1309	0	80	FEMALE	G2	Stage II	T2	unknow	N0
TCGA-BB-A5HZ	827	0	65	MALE	G2	Stage IVA	T4a	M0	N1
TCGA-BA-5149	806	1	47	MALE	G2	Stage IVA	T3	unknow	N2c
TCGA-HD-7831	667	0	74	MALE	G2	Stage IVA	T2	unknow	N2
TCGA-CV-7235	2347	0	67	MALE	G3	Stage II	T2	unknow	N0
TCGA-CV-A468	464	1	42	MALE	G2	Stage IVA	T4a	M0	N2a
TCGA-UF-A71E	1504	1	63	MALE	G3	Stage IVA	T4a	M0	N0
TCGA-CR-7386	1430	0	69	MALE	G1	Stage IVA	T4a	M0	N2c
TCGA-UP-A6WW	518	0	58	MALE	G2	unknow	unknow	unknow	unknow
TCGA-UF-A719	1663	0	54	MALE	G1	Stage II	T2	M0	N0
TCGA-DQ-7592	1143	0	57	MALE	G2	unknow	TX	unknow	NX
TCGA-CR-7395	930	0	80	FEMALE	G2	Stage II	T2	M0	N0
TCGA-CV-7100	274	1	66	MALE	G3	Stage III	T2	unknow	N1
TCGA-CV-6951	915	1	57	MALE	G2	Stage IVA	T4a	unknow	N2c
TCGA-CQ-5326	89	1	67	MALE	G3	Stage IVA	T4a	unknow	N2c
TCGA-CQ-5334	129	1	87	MALE	G2	Stage IVA	T3	unknow	N2b
TCGA-CV-5973	2641	0	62	FEMALE	G3	Stage III	T3	unknow	N1
TCGA-CV-7253	361	1	58	MALE	G2	Stage IVA	T4a	unknow	N0
TCGA-CV-7254	1459	1	55	MALE	G3	Stage II	T2	unknow	NX
TCGA-CR-7373	889	0	66	MALE	G2	Stage IVA	T4a	M0	N1
TCGA-CQ-7063	2133	0	59	FEMALE	G1	Stage I	T1	M0	NX
TCGA-CV-6961	76	1	61	MALE	G3	Stage II	T2	unknow	N0
TCGA-D6-A6EN	687	0	71	MALE	G3	Stage III	T2	M0	N1
TCGA-CV-A45O	851	0	57	MALE	G2	unknow	unknow	unknow	unknow
TCGA-F7-A61S	576	0	62	MALE	G1	Stage III	T3	M0	N0
TCGA-CV-A45P	639	0	82	FEMALE	G2	Stage I	T1	M0	N0
TCGA-CN-4742	397	1	48	FEMALE	G3	Stage IVA	T4a	M0	N2b
TCGA-KU-A6H8	327	1	41	MALE	G3	Stage IVA	T2	M0	N2b

TCGA-RS-A6TO	387	1	82	FEMALE	G2	Stage IVA	T4	M0	N2c
TCGA-CN-5364	493	1	55	MALE	G2	Stage IVA	T4a	M0	N2c
TCGA-CV-7423	3059	1	65	MALE	G1	Stage II	T2	unknow	NX
TCGA-CQ-A4CG	430	1	78	FEMALE	G2	Stage III	T3	M0	N0
TCGA-CV-7102	56	1	76	FEMALE	G3	Stage IVA	T3	unknow	N2
TCGA-CV-A463	23	1	82	FEMALE	G2	Stage IVA	T4a	M0	N0
TCGA-CV-7103	1591	1	49	MALE	G2	Stage IVA	T2	unknow	N2b
TCGA-CN-A642	82	1	57	MALE	G3	Stage IVB	T4a	M0	N3
TCGA-BA-5152	1288	0	56	MALE	G2	Stage IVA	T4a	M0	N0
TCGA-CV-A45V	32	1	87	FEMALE	G1	Stage IVA	T4	M0	N0
TCGA-HD-8635	695	0	61	FEMALE	G2	Stage III	T1	MX	N1
TCGA-QK-AA3K	253	0	60	MALE	G2	Stage IVA	T3	MX	N2b
TCGA-H7-7774	407	0	75	FEMALE	G2	Stage IVA	T4a	unknow	N0
TCGA-QK-A8Z9	449	1	56	MALE	G2	Stage IVA	T4a	M0	N2b
TCGA-CR-7367	1440	0	52	MALE	G1	Stage IVA	T4a	M0	N1
TCGA-CV-7413	294	1	74	FEMALE	G2	Stage II	T2	unknow	N0
TCGA-CN-4741	2239	0	75	MALE	G2	Stage IVA	T4a	M0	N0
TCGA-T2-A6X2	987	0	82	MALE	G1	Stage III	T3	MX	N0
TCGA-CV-6952	185	1	65	FEMALE	G1	Stage IVA	T3	unknow	N2b
TCGA-CV-7434	218	1	64	MALE	G2	Stage IVA	T4a	unknow	N1
TCGA-CQ-5324	1593	0	59	MALE	G2	Stage III	T3	unknow	N0
TCGA-WA-A7GZ	625	1	58	MALE	G2	unknow	T2	unknow	N0
TCGA-CQ-7071	1311	0	76	FEMALE	G2	Stage III	T2	M0	N1
TCGA-C9-A480	386	0	45	FEMALE	G1	Stage III	T3	M0	N0
TCGA-CV-A6JN	906	0	53	MALE	G1	unknow	unknow	unknow	unknow
TCGA-UF-A71B	1506	0	50	MALE	G2	Stage IVA	T4	M0	N0
TCGA-CV-6938	144	1	87	MALE	G1	Stage II	T2	unknow	NX
TCGA-CV-6942	4282	0	73	FEMALE	G3	Stage II	T2	unknow	NX
TCGA-IQ-A6SH	471	0	55	MALE	G1	Stage III	T2	M0	N1
TCGA-IQ-A61L	416	0	72	FEMALE	G1	Stage II	T2	M0	N0
TCGA-CV-7238	2727	0	69	FEMALE	G2	Stage II	T2	unknow	N0
TCGA-CV-7252	151	1	62	FEMALE	G2	Stage IVA	T4a	unknow	N0
TCGA-CN-4728	1724	0	56	MALE	G2	Stage IVA	T3	unknow	N2b
TCGA-CN-A63V	679	0	59	MALE	G2	Stage IVA	T4a	MX	N0
TCGA-IQ-7631	1172	0	60	FEMALE	G1	Stage II	T2	unknow	N0
TCGA-CQ-A4C9	707	0	56	MALE	G2	Stage III	T2	M0	N1
TCGA-BA-5151	722	0	72	MALE	G1	Stage IVA	T4a	unknow	N0
TCGA-CQ-6219	479	1	50	FEMALE	G2	Stage IVA	T3	unknow	N2a
TCGA-CR-6488	379	0	68	FEMALE	G2	Stage II	T2	unknow	N0
TCGA-CR-7377	279	1	58	MALE	G3	Stage IVA	T4a	M0	N2c
TCGA-CQ-5333	341	1	74	MALE	G3	Stage II	T2	unknow	N0
TCGA-D6-8569	770	0	52	MALE	G2	Stage II	T2	M0	N0
TCGA-C9-A47Z	191	1	72	FEMALE	G1	Stage III	T2	M0	N1
TCGA-CV-6934	65	1	66	FEMALE	G2	Stage IVA	T3	unknow	N2b

TCGA-HD-A633	421	0	74	MALE	G2	Stage IVA	T4a	MX	N0
TCGA-F7-8489	658	0	48	MALE	G1	Stage II	T2	M0	N0
TCGA-F7-A61W	14	0	51	MALE	G2	Stage IVA	T2	M0	N2b
TCGA-CV-A465	215	1	24	MALE	G1	Stage III	T3	M0	N0
TCGA-CQ-6228	456	1	71	FEMALE	G2	Stage IVA	T1	unknow	N2b
TCGA-CQ-6222	2016	0	63	MALE	G2	Stage IVA	T2	unknow	N2b
TCGA-QK-A64Z	641	1	79	FEMALE	G1	Stage IVA	T4	MX	NX
TCGA-CV-6960	862	1	49	MALE	G3	Stage III	T3	unknow	N0
TCGA-IQ-A61J	1021	0	54	MALE	G1	Stage IVA	T2	M0	N2b
TCGA-CN-4734	1690	0	70	MALE	G2	Stage II	T2	unknow	N0
TCGA-CV-6954	2002	1	59	MALE	G2	Stage IVA	T4a	unknow	NX
TCGA-CN-6019	1038	0	61	MALE	G2	Stage IVA	T4a	unknow	N0
TCGA-P3-A5QA	2182	0	41	MALE	G3	Stage I	T1	MX	NX
TCGA-CV-7446	1093	1	66	MALE	G2	Stage IVA	T2	unknow	N2b
TCGA-CR-7393	993	0	26	MALE	G2	Stage III	T1	M0	N1
TCGA-CN-6017	853	1	55	MALE	G2	Stage IVA	T3	unknow	N2b
TCGA-P3-A6T0	578	0	47	FEMALE	G2	Stage IVA	T4a	MX	N0
TCGA-CN-A640	134	1	40	FEMALE	G2	Stage IVA	T2	MX	N2b
TCGA-DQ-5625	1133	1	52	FEMALE	G2	unknow	TX	unknow	NX
TCGA-CQ-6227	129	1	77	MALE	G2	Stage IVA	T3	unknow	N2c
TCGA-CV-7178	2166	1	64	FEMALE	G3	Stage IVA	T4a	unknow	N2
TCGA-P3-A6T3	577	1	49	MALE	G2	Stage IVA	T3	MX	N2
TCGA-CQ-7065	1628	0	40	MALE	G2	Stage II	T2	unknow	N0
TCGA-CN-4731	998	1	63	FEMALE	G3	Stage IVA	T4a	unknow	N2c
TCGA-CV-A6K0	606	0	58	MALE	G3	Stage I	T1	M0	N0
TCGA-CQ-A4CD	1022	0	69	MALE	G3	Stage IVA	T4a	M0	N0
TCGA-CN-5359	377	1	59	MALE	G2	Stage IVA	T4a	M0	N2b
TCGA-H7-A6C4	414	0	35	FEMALE	G2	Stage IVA	T2	M0	N2b
TCGA-CQ-6218	1253	0	52	FEMALE	G2	Stage IVA	T3	unknow	N2b
TCGA-UF-A71A	86	1	67	MALE	G1	Stage IVA	T4a	M0	N2c
TCGA-CV-A6K2	317	1	79	MALE	G1	Stage IVA	T4	M0	N0
TCGA-T2-A6WX	209	1	73	FEMALE	G1	unknow	unknow	unknow	unknow
TCGA-CQ-6225	403	1	65	MALE	G2	Stage III	T3	unknow	N0
TCGA-P3-A6T7	487	1	55	MALE	G2	Stage IVA	T3	MX	N2b
TCGA-CX-A4AQ	1555	0	56	MALE	G3	Stage IVA	T2	M0	N2b
TCGA-CV-6940	804	1	80	FEMALE	G2	Stage III	T2	unknow	N1
TCGA-CR-7376	972	0	83	MALE	G2	Stage III	T2	M0	N1
TCGA-IQ-A6SG	579	0	61	FEMALE	G2	Stage III	T3	M0	N0
TCGA-CQ-A4C7	353	1	88	MALE	G3	Stage III	T3	M0	N1
TCGA-MT-A67A	914	0	85	FEMALE	G2	Stage I	T1	MX	N0
TCGA-CN-4729	392	0	73	MALE	G2	Stage III	T2	unknow	N1
TCGA-CV-7099	243	1	85	FEMALE	G2	Stage II	T2	unknow	NX
TCGA-CV-A45R	5480	0	46	MALE	G1	Stage III	T2	M0	N1
TCGA-CN-4740	839	1	79	FEMALE	G2	Stage IVA	T4a	M0	N0

TCGA-CX-7082	11	1	82	MALE	G2	Stage IVA	T2	unknow	N2b
TCGA-CR-7397	754	0	44	MALE	G2	Stage IVA	T3	M0	N2b
TCGA-QK-A6IG	222	1	69	MALE	G2	Stage III	T2	M0	N1
TCGA-CV-6441	292	1	60	MALE	G3	Stage III	T3	unknow	N0
TCGA-CQ-6224	1721	0	52	MALE	G3	Stage IVA	T2	unknow	N2b
TCGA-CV-5970	406	1	59	MALE	G2	Stage IVA	T4a	unknow	N2b
TCGA-CV-7180	327	1	34	MALE	G2	Stage II	T2	unknow	NX
TCGA-BA-A6DJ	407	1	62	MALE	G2	Stage IVA	T4a	M0	N2c
TCGA-BA-6873	122	0	28	MALE	G2	Stage IVA	T4a	unknow	N2b
TCGA-UF-A7JA	2265	0	66	FEMALE	G2	Stage IVA	T4a	M0	N1
TCGA-CV-7438	194	1	87	FEMALE	G3	Stage I	T1	unknow	NX
TCGA-CV-A6JO	197	1	69	MALE	G2	Stage IVA	T3	M0	N2c
TCGA-CV-A6JE	1075	0	78	MALE	G2	unknow	unknow	unknow	unknow
TCGA-CN-6998	357	1	53	MALE	G2	Stage IVA	T3	unknow	N2b
TCGA-BA-A6D8	850	0	59	MALE	G2	Stage IVA	T4a	M0	N2c
TCGA-CX-7219	1045	0	47	MALE	G2	Stage IVA	T4a	unknow	N2c
TCGA-CV-7243	954	0	50	MALE	G2	Stage III	T2	unknow	N1
TCGA-D6-A6EM	232	0	65	FEMALE	G2	Stage III	T2	M0	N1
TCGA-DQ-7588	427	1	66	MALE	G3	unknow	TX	unknow	NX
TCGA-CR-7391	913	0	36	FEMALE	G1	Stage I	T1	M0	N0
TCGA-QK-A8Z7	392	0	59	MALE	unknow	Stage IVA	T4a	M0	N2c
TCGA-CV-7414	14	1	78	MALE	G2	Stage IVA	T4a	unknow	N2b
TCGA-BA-5557	623	0	41	FEMALE	G2	Stage III	T1	unknow	N1
TCGA-CN-6018	580	1	85	FEMALE	G2	Stage IVA	T4a	unknow	N2b
TCGA-HD-A6HZ	111	0	79	FEMALE	G2	Stage III	T2	MX	N1
TCGA-BB-7872	1168	0	63	MALE	G3	Stage I	T1	unknow	N0
TCGA-CR-6492	479	0	78	MALE	G3	Stage III	T2	M0	N1
TCGA-CQ-6221	1000	0	79	MALE	G3	unknow	T2	unknow	NX
TCGA-F7-A624	378	0	73	MALE	G3	Stage II	T2	M0	NX
TCGA-CV-A464	1722	0	48	MALE	G2	Stage III	T3	M0	N0
TCGA-UF-A7JD	739	1	71	MALE	G3	Stage IVA	T4	M0	N2b
TCGA-DQ-5624	1778	0	43	FEMALE	G2	unknow	TX	unknow	NX
TCGA-CN-A498	773	1	61	FEMALE	G1	Stage III	T3	MX	N0
TCGA-CV-7091	3381	0	54	MALE	G3	Stage I	T1	unknow	N0
TCGA-HL-7533	1057	0	65	MALE	G3	unknow	unknow	unknow	unknow
TCGA-CR-6493	282	1	69	MALE	G2	Stage IVA	T3	unknow	N2b
TCGA-CR-6491	693	0	60	MALE	G2	Stage IVA	T4a	M0	N2b
TCGA-CV-A45X	198	1	47	MALE	G2	Stage IVA	T2	M0	N2b
TCGA-CN-4736	395	1	70	FEMALE	G2	unknow	T1	unknow	NX
TCGA-CQ-A4C6	1353	0	63	MALE	G2	Stage II	T2	M0	N0
TCGA-CQ-7072	2359	0	51	MALE	G3	Stage II	T2	M0	N0
TCGA-D6-6827	568	0	55	FEMALE	G3	Stage I	T1	unknow	N0
TCGA-IQ-A61E	1147	0	55	FEMALE	G2	Stage III	T3	MX	N0
TCGA-BA-A6DG	69	1	49	MALE	G2	unknow	TX	MX	NX

TCGA-QK-A6IJ	387	0	71	MALE	G3	Stage III	T2	M0	N1
TCGA-CV-A6JT	852	0	65	MALE	G2	Stage II	T2	M0	N0
TCGA-CQ-A4CH	379	1	58	MALE	G2	Stage II	T2	M0	N0
TCGA-CV-A45Q	5152	1	69	FEMALE	G1	unknow	unknow	unknow	unknow
TCGA-BA-A6DB	216	0	24	FEMALE	G1	Stage I	T1	M0	N0
TCGA-CN-6013	727	1	56	MALE	G3	Stage IVA	T4a	unknow	N2b
TCGA-CR-7372	759	0	45	MALE	G1	Stage I	T1	M0	N0
TCGA-CQ-5327	1660	0	61	FEMALE	G2	Stage IVA	T3	unknow	N2c
TCGA-D6-6825	491	0	73	MALE	G2	Stage I	T1	unknow	N0
TCGA-BA-6872	384	1	47	MALE	G2	unknow	TX	unknow	NX
TCGA-CQ-5330	1897	0	69	FEMALE	G3	Stage IVA	T3	unknow	N2b
TCGA-QK-A652	645	0	60	MALE	G2	Stage III	T1	MX	N1
TCGA-BB-8601	624	0	84	MALE	G2	Stage III	T3	MX	N1
TCGA-CN-4737	625	0	19	MALE	G2	Stage IVA	T2	unknow	N2b
TCGA-P3-A6T6	395	1	53	MALE	G3	Stage IVA	T4a	MX	NX
TCGA-CV-6436	1899	0	62	MALE	G1	Stage IVA	T4a	unknow	N0
TCGA-BA-7269	1273	0	61	MALE	G1	Stage III	T2	unknow	N1
TCGA-CN-5373	1584	0	55	FEMALE	G1	Stage I	T1	unknow	N0
TCGA-D6-A4ZB	376	0	61	MALE	G2	Stage III	T3	M0	N0
TCGA-BA-4075	283	1	49	MALE	G2	Stage III	T3	M0	N0
TCGA-CN-6011	933	0	57	MALE	G3	Stage IVA	T4a	unknow	N0
TCGA-CV-6939	666	1	60	MALE	G3	Stage IVA	T4a	unknow	N2b
TCGA-BB-A5HU	782	0	47	MALE	G2	Stage IVA	T4	M0	N2b
TCGA-CV-7263	560	1	64	MALE	G1	Stage II	T2	unknow	NX
TCGA-MT-A67D	56	0	55	MALE	G2	Stage II	T2	M0	N0
TCGA-HD-7917	836	1	62	MALE	G1	Stage II	T2	unknow	N0
TCGA-D6-A6EO	759	0	44	MALE	G2	Stage IVA	T4a	M0	N0
TCGA-CR-7382	796	0	49	MALE	G2	Stage IVA	T2	M0	N2c
TCGA-CV-6936	166	1	68	MALE	G2	Stage IVA	T4a	unknow	N2c
TCGA-CV-7407	1081	1	67	FEMALE	G2	Stage II	T2	unknow	NX
TCGA-DQ-5631	548	1	52	MALE	G3	unknow	TX	unknow	NX
TCGA-CN-5369	1	1	90	FEMALE	G3	Stage IVA	T4a	M0	N0
TCGA-BA-A6DD	173	1	44	MALE	G2	Stage IVA	T4a	M0	N2c
TCGA-CV-6955	334	1	74	FEMALE	G3	Stage II	T2	unknow	NX
TCGA-IQ-A61G	360	0	57	MALE	G2	Stage IVA	T4a	MX	N2c
TCGA-CV-A6JY	646	0	69	MALE	G1	Stage IVA	T4a	M0	N0
TCGA-CQ-6229	1179	0	61	MALE	G2	Stage II	T2	unknow	N0
TCGA-CQ-6220	985	1	69	MALE	G2	Stage III	T3	unknow	N0
TCGA-CV-7411	2717	1	64	FEMALE	G1	Stage IVA	T4a	unknow	N0
TCGA-BA-A6DF	238	1	80	FEMALE	G2	Stage IVA	T4a	M0	N0
TCGA-CR-7394	1346	0	70	MALE	G2	Stage IVA	T4a	M0	N0
TCGA-CN-A49A	526	1	60	MALE	G2	Stage IVB	T4b	MX	N0
TCGA-P3-A6T8	400	0	54	MALE	G3	Stage IVA	T4a	MX	N2
TCGA-IQ-A61H	1138	0	76	MALE	G2	Stage II	T2	MX	N0

TCGA-CQ-5329	2143	0	46	FEMALE	G2	Stage II	T2	unknow	N0
TCGA-D6-6515	403	1	82	FEMALE	G3	Stage II	T2	unknow	N0
TCGA-CN-6994	1183	0	67	MALE	G2	Stage IVA	T4a	unknow	N0
TCGA-CR-7380	606	1	58	MALE	G2	Stage IVB	T4b	unknow	N0
TCGA-UF-A7JO	631	1	79	FEMALE	G2	Stage IVA	T4a	M0	N0
TCGA-CV-7095	572	1	87	FEMALE	G2	Stage II	T2	unknow	NX
TCGA-CV-A6JZ	714	0	68	MALE	G2	Stage II	T2	M0	N0
TCGA-CV-7090	5252	0	39	MALE	G2	Stage II	T2	unknow	NX
TCGA-CR-6484	354	0	67	FEMALE	G2	Stage IVA	T4a	M0	N2
TCGA-CN-6020	205	1	58	MALE	G2	Stage III	T2	unknow	N1
TCGA-CV-6945	366	1	41	MALE	G2	Stage IVA	T4a	unknow	N2
TCGA-BA-4077	1134	1	45	FEMALE	G2	Stage IVA	T4a	M0	N0
TCGA-CV-7097	385	1	53	MALE	G3	Stage II	T2	unknow	NX
TCGA-CQ-5331	1399	0	73	FEMALE	G2	unknow	T2	unknow	NX
TCGA-CN-5358	261	1	60	MALE	G2	Stage II	T2	unknow	N0
TCGA-KU-A66T	552	0	53	FEMALE	G2	Stage IVA	T4	MX	N0
TCGA-P3-A5QF	330	1	49	MALE	G2	Stage IVA	T4	MX	N2b
TCGA-T3-A92N	95	1	79	MALE	G3	Stage IVA	T2	MX	N2c
TCGA-HD-7832	836	0	52	MALE	G2	Stage IVA	T4a	unknow	N0
TCGA-P3-A6T2	2298	0	45	MALE	G2	Stage IVA	T3	MX	N2b
TCGA-IQ-7632	441	0	68	FEMALE	G1	Stage IVA	T4a	M0	N0
TCGA-F7-A50G	616	0	66	MALE	G1	Stage III	T3	M0	N1
TCGA-CR-7392	1425	0	67	FEMALE	G1	Stage IVA	T3	M0	N2b
TCGA-CQ-5323	1466	0	82	MALE	G2	Stage I	T1	unknow	N0
TCGA-HD-A6I0	210	0	56	MALE	G1	Stage IVA	T4a	MX	N1
TCGA-CV-6953	1641	1	80	FEMALE	G1	Stage III	T3	unknow	N0
TCGA-P3-A6T4	62	1	54	MALE	G2	Stage IVA	T4a	MX	N1
TCGA-CV-6003	1665	0	50	FEMALE	G2	Stage III	T2	unknow	N1
TCGA-CV-5979	1315	0	26	MALE	G2	Stage IVA	T2	unknow	N2b
TCGA-BB-4224	278	0	52	MALE	G2	Stage IVA	T2	unknow	N2b
TCGA-CV-A6JM	194	1	85	MALE	G2	unknow	unknow	unknow	unknow
TCGA-CV-6941	342	1	51	MALE	G2	Stage III	T3	unknow	N0
TCGA-CN-6024	337	1	66	MALE	G2	Stage IVA	T4a	M0	N2c
TCGA-CN-6016	1443	0	64	MALE	G2	Stage IVA	T4a	unknow	N1
TCGA-BB-A6UO	268	1	61	FEMALE	G2	Stage IVA	T4a	MX	N2b
TCGA-CR-7368	1245	0	54	MALE	G2	Stage IVA	T4a	M0	N1
TCGA-CQ-5325	654	1	65	MALE	G2	Stage I	T1	unknow	N0
TCGA-CV-A6JD	182	1	82	FEMALE	G3	unknow	unknow	unknow	unknow
TCGA-CQ-A4CA		0	unknow	MALE	G2	Stage II	T2	M0	N0
TCGA-CV-6959	256	1	48	MALE	G2	Stage III	T3	unknow	N0
TCGA-CV-7428	1671	1	47	MALE	G2	Stage IVA	T4a	unknow	N2b
TCGA-CV-7429	107	1	55	MALE	G3	Stage IVA	T4a	unknow	N2b
TCGA-CV-7427	4760	1	73	FEMALE	G1	Stage II	T2	unknow	NX
TCGA-UF-A7JT	993	1	72	FEMALE	G3	Stage IVA	T4a	M0	N0

TCGA-MT-A67F	384	0	60	FEMALE	G2	Stage IVA	T4	MX	N0
TCGA-CR-7365	1191	0	60	MALE	G2	Stage IVA	T4a	M0	N0
TCGA-F7-A50J	947	0	67	FEMALE	G2	Stage III	T3	M0	N0
TCGA-CN-5370	259	1	78	MALE	G3	Stage III	T3	M0	N1
TCGA-CV-7104	393	1	61	FEMALE	G2	Stage IVA	T2	unknow	N2b
TCGA-HD-A4C1	11	0	41	FEMALE	G1	Stage IVA	T4a	MX	N2b
TCGA-UF-A7JC	546	1	42	MALE	G1	Stage IVA	T3	M0	N2b
TCGA-CN-5367	352	1	60	FEMALE	G2	Stage IVA	T4a	M0	N2b
TCGA-CV-7409	543	1	43	MALE	G3	Stage IVB	T4a	unknow	N3
TCGA-CV-6933	2741	1	53	MALE	G2	Stage III	T3	unknow	N1
TCGA-BA-4074	462	1	69	MALE	G3	Stage IVA	T2	M0	N2c
TCGA-H7-8502	458	0	50	MALE	G2	Stage IVA	T4a	M0	N2b
TCGA-CN-4726	142	1	68	MALE	G2	Stage IVA	T3	unknow	N2b
TCGA-CV-5442	2327	0	76	FEMALE	G3	Stage IVA	T4a	unknow	N2b
TCGA-CV-5977	1840	0	66	MALE	G2	Stage IVA	T3	unknow	N2b
TCGA-CV-6937	624	1	71	MALE	G2	Stage II	T2	unknow	N0
TCGA-CQ-5332	317	1	87	MALE	G2	Stage III	T3	unknow	N0
TCGA-CV-6956	217	1	67	MALE	G2	Stage III	T3	unknow	N1
TCGA-H7-8501	461	0	54	MALE	GX	Stage IVA	T4a	M0	N1

Supplementary table 3 Univariate cox regression of overall survival and clinicopathologic characteristics in 30 pairs OSCC clinical samples.

Clinical characteristics	Hazard ratio	HR (95% CI)	*p*-value
Age (≤65 vs. >65)	0.9968	0.9529-1.0428	0.89160
Gender (Female vs. male)	1.0666	0.3315-3.4314	0.91379
Clinical stage (I/II/III/IV)	2.1956	1.0282-4.6885	0.04217*
T stage (T1/2/3/4)	2.2025	1.0793-4.4946	0.03002*
N stage(N0/1/2)	2.7477	1.3124-5.7525	0.00734*
IGF2BP2 expression(low/high)	1.0939	1.024-1.1682	0.00743*

*$p < 0.05$ was considered statistically significant.

Supplementary table 4 Multivariate analyses of overall survival and clinicopathologic characteristics in 30 pairs Clinical OSCC patients.

Clinical characteristics	Hazard ratio	HR (95% CI)	*p*-value
N stage(N0/1/2)	2.5477	1.1568-5.6109	0.02025*
IGF2BP2 expression(low/high)	1.0881	1.0171-1.1639	0.01412*

*$p < 0.05$ was considered statistically significant.

Supplementary table 5 KEGG pathway enrichment analysis results of IGF2BP2 related genes.

ID	pvalue	qvalue	geneID
hsa04512	0.000369	0.011980851	COL4A6/LAMC2/ITGA6/ITGA5
hsa04151	0.000372	0.011980851	CDK6/COL4A6/LAMC2/TGFA/ITGA6/ITGA5/EGFR
hsa05222	0.000438	0.011980851	CDK6/COL4A6/LAMC2/ITGA6
hsa04510	0.00104	0.02134544	COL4A6/LAMC2/ITGA6/ITGA5/EGFR
hsa05165	0.001629	0.026758044	CDK6/COL4A6/LAMC2/ITGA6/ITGA5/EGFR
hsa05223	0.002769	0.029585268	CDK6/TGFA/EGFR
hsa05214	0.00311	0.029585268	CDK6/TGFA/EGFR
hsa05212	0.003229	0.029585268	CDK6/TGFA/EGFR
hsa05412	0.003351	0.029585268	DSG2/ITGA6/ITGA5
hsa01521	0.003603	0.029585268	NRG1/TGFA/EGFR
hsa04012	0.004429	0.033059244	NRG1/TGFA/EGFR
hsa00601	0.004981	0.034078745	B3GNT5/B3GNT3
hsa04350	0.005868	0.037063074	FST/INHBA/ID2
hsa04640	0.006776	0.037291225	CSF2/ITGA6/ITGA5
hsa05206	0.006813	0.037291225	HMGA2/CDK6/ITGA5/EGFR/IGF2BP1
hsa05146	0.007358	0.037759863	COL4A6/LAMC2/CSF2

Supplementary table 6 GO biological function enrichment analysis results of IGF2BP2 related genes.

ID	pvalue	qvalue	geneID
GO:0030198	7.03E-08	4.30E-05	PHLDB2/COL4A6/LAMC2/ITGA6/ITGA5/SERPINE1/FBLN1/DPT/TPSAB1/ELN
GO:0043062	7.20E-08	4.30E-05	PHLDB2/COL4A6/LAMC2/ITGA6/ITGA5/SERPINE1/FBLN1/DPT/TPSAB1/ELN
GO:0003161	4.92E-06	0.001525	DSG2/NRG1/ID2
GO:0021537	5.12E-06	0.001525	CDK6/NRG1/EGFR/IGF2BP1/INHBA/ID2/AQP1
GO:0007569	1.49E-05	0.003548	HMGA2/CDK6/SERPINE1/PRELP/ID2
GO:0010810	2.25E-05	0.004357	PHLDB2/CDK6/ITGA6/ITGA5/SERPINE1/FBLN1
GO:0042476	2.56E-05	0.004357	FST/ITGA6/SERPINE1/INHBA/AQP1
GO:2001236	6.81E-05	0.008602	CSF2/ITGA6/SERPINE1/INHBA/EYA2
GO:0048708	6.88E-05	0.008602	CDK6/EGFR/S100B/ID2
GO:0030900	7.21E-05	0.008602	CDK6/NRG1/EGFR/IGF2BP1/INHBA/ID2/AQP1
GO:0048333	0.000108	0.011708	HMGA2/INHBA/EYA2
GO:0007044	0.000137	0.012182	PHLDB2/LAMC2/ITGA6/ITGA5
GO:0007369	0.000141	0.012182	HMGA2/PHLDB2/ITGA5/INHBA/EYA2
GO:0048511	0.000148	0.012182	CSF2/EGFR/SERPINE1/INHBA/PTGDS/ID2
GO:0150115	0.000153	0.012182	PHLDB2/LAMC2/ITGA6/ITGA5
GO:2001237	0.000184	0.013735	CSF2/ITGA6/SERPINE1/EYA2
GO:0002573	0.00023	0.014195	CDK6/CSF2/INHBA/CCL19/ID2
GO:0035987	0.000245	0.014195	HMGA2/ITGA5/INHBA
GO:0030225	0.000262	0.014195	CSF2/INHBA/ID2
GO:0007623	0.000273	0.014195	CSF2/EGFR/SERPINE1/PTGDS/ID2
GO:2001239	0.000279	0.014195	CSF2/INHBA/EYA2
GO:0048512	0.000296	0.014195	CSF2/PTGDS/ID2
GO:0021782	0.000305	0.014195	CDK6/NRG1/EGFR/ID2
GO:0007622	0.000314	0.014195	CSF2/PTGDS/ID2
GO:0045646	0.000314	0.014195	CDK6/INHBA/ID2
GO:0048146	0.000334	0.014195	CDK6/EGFR/AQP1
GO:0010001	0.000349	0.014195	CDK6/NRG1/EGFR/S100B/ID2
GO:0097191	0.000349	0.014195	CSF2/ITGA6/SERPINE1/INHBA/EYA2
GO:0001704	0.000355	0.014195	HMGA2/ITGA5/INHBA/EYA2
GO:0031589	0.000357	0.014195	PHLDB2/CDK6/ITGA6/ITGA5/SERPINE1/FBLN1
GO:0001706	0.000417	0.014798	HMGA2/ITGA5/INHBA
GO:0003229	0.000417	0.014798	DSG2/NRG1/ID2
GO:0018108	0.000444	0.014798	NRG1/CSF2/TGFA/ITGA5/EGFR/EPHB6
GO:0010755	0.000447	0.014798	SERPINE1/CLEC3B
GO:0021670	0.000447	0.014798	CDK6/AQP1
GO:0031581	0.000447	0.014798	LAMC2/ITGA6
GO:0018212	0.000463	0.01492	NRG1/CSF2/TGFA/ITGA5/EGFR/EPHB6
GO:0061029	0.000527	0.01654	EGFR/INHBA
GO:0014706	0.000546	0.016698	DSG2/NRG1/S100B/EYA2/ID2/ELN

GO:0050730	0.000643	0.019184	NRG1/CSF2/TGFA/ITGA5/EGFR
GO:0032274	0.000707	0.020163	FOXD1/INHBA
GO:0060537	0.00071	0.020163	DSG2/NRG1/S100B/EYA2/ID2/ELN
GO:0070613	0.000745	0.020654	SERPINE1/CST7/CLEC3B
GO:0010812	0.000778	0.021038	PHLDB2/SERPINE1/FBLN1
GO:0042035	0.000807	0.021038	IGF2BP3/IGF2BP1
GO:1903317	0.000811	0.021038	SERPINE1/CST7/CLEC3B
GO:0035313	0.001025	0.024184	PHLDB2/ITGA5
GO:0045187	0.001025	0.024184	CSF2/PTGDS
GO:0001707	0.001034	0.024184	HMGA2/INHBA/EYA2
GO:0038034	0.001034	0.024184	CSF2/INHBA/EYA2
GO:0097192	0.001034	0.024184	CSF2/INHBA/EYA2
GO:0048332	0.001116	0.025545	HMGA2/INHBA/EYA2
GO:0031643	0.001144	0.025545	CST7/S100B
GO:0050890	0.001195	0.025545	ITGA5/EGFR/S100B/CHL1/CBR3
GO:0007492	0.001202	0.025545	HMGA2/ITGA5/INHBA
GO:0090398	0.001202	0.025545	HMGA2/CDK6/ID2
GO:0051897	0.001258	0.025545	NRG1/TGFA/EGFR/CCL19
GO:0042089	0.001269	0.025545	IGF2BP3/IGF2BP1
GO:0071731	0.001269	0.025545	CCL19/AQP1
GO:0042063	0.001285	0.025545	CDK6/NRG1/EGFR/S100B/ID2
GO:0048145	0.001386	0.026926	CDK6/EGFR/AQP1
GO:0042107	0.0014	0.026926	IGF2BP3/IGF2BP1
GO:0048144	0.001435	0.027167	CDK6/EGFR/AQP1
GO:0007568	0.001522	0.027685	HMGA2/CDK6/SERPINE1/PRELP/ID2
GO:0042749	0.001537	0.027685	CSF2/PTGDS
GO:0050802	0.001537	0.027685	CSF2/PTGDS
GO:0008544	0.001568	0.027685	DSG2/PTHLH/LAMC2/FST/EGFR/INHBA
GO:0001942	0.001588	0.027685	FST/EGFR/INHBA
GO:0031639	0.001681	0.027685	SERPINE1/CLEC3B
GO:0045649	0.001681	0.027685	INHBA/ID2
GO:2000773	0.001681	0.027685	HMGA2/CDK6
GO:0022404	0.001695	0.027685	FST/EGFR/INHBA
GO:0022405	0.001695	0.027685	FST/EGFR/INHBA
GO:0098773	0.00175	0.027956	FST/EGFR/INHBA
GO:0050731	0.001758	0.027956	NRG1/CSF2/TGFA/ITGA5
GO:0007565	0.001791	0.027974	DSG2/PTHLH/ITGA5/FBLN1
GO:0042475	0.001806	0.027974	FST/ITGA6/SERPINE1
GO:1901654	0.001928	0.029482	DSG2/EGFR/CCL19/AQP1
GO:0022410	0.001986	0.029985	CSF2/PTGDS
GO:0071902	0.002144	0.031628	HMGA2/NRG1/TGFA/EGFR/CCL19
GO:0070633	0.002148	0.031628	CSF2/AQP1
GO:0045766	0.002224	0.032341	HMGA2/ITGA5/SERPINE1/AQP1
GO:0018146	0.00249	0.034936	B3GNT3/PRELP

GO:0042044	0.00249	0.034936	CSF2/AQP1
GO:0042745	0.00249	0.034936	CSF2/PTGDS
GO:0045577	0.00267	0.036388	INHBA/ID2
GO:0090344	0.00267	0.036388	HMGA2/CDK6
GO:1902106	0.002715	0.036388	CDK6/INHBA/ID2
GO:2001022	0.002715	0.036388	HMGA2/EGFR/EYA2
GO:0062023	1.04E-07	1.15E-05	COL4A6/LAMC2/ITGA6/SERPINE1/FBLN1/PRELP/DPT/TPSAB1/ELN/CLEC3B
GO:0045178	8.83E-07	4.88E-05	PHLDB2/SLC7A5/ITGA6/EGFR/AQP1
GO:0009925	8.64E-06	0.000318	SLC7A5/ITGA6/EGFR/AQP1
GO:0005604	0.000139	0.003831	COL4A6/LAMC2/ITGA6/FBLN1
GO:0016323	0.000404	0.008921	SLC7A5/TGFA/ITGA6/EGFR/AQP1
GO:0005201	5.62E-06	0.000846	COL4A6/LAMC2/FBLN1/PRELP/DPT/ELN
